# TACO1 regulates mitochondrial adaptation in hypertension-induced cardiac remodeling and heart failure

**DOI:** 10.21203/rs.3.rs-9589283/v1

**Published:** 2026-05-05

**Authors:** Berwin Singh Swami Vetha, Ronald McMillan, James Marchant, Mohd Mabood Khan, Joyonna Gamble-George, Jeremiah Afolabi, Edgar Garza-Lopez, Andrea G. Marshall, Calixto Pablo Hernandez Perez, Dillon Garbrandt, Bret Mobley, Jenny Schafer, Max Kushner, Oleg Kovtun, Debra D. Murray, Tonya Zeczycki, Annet Kirabo, Alexandre Colas, Celestine Wanjalla, Dao-Fu Dai, Melanie McReynolds, Azeez Aileru, Antentor Hinton

**Affiliations:** 1 Department of Foundational Science, East Carolina University, Greenville, NC, USA; 2 Department of Pharmacology and Toxicology, East Carolina University, Greenville, NC, USA; 3 Department of Medicine, Division of Infectious Diseases, Vanderbilt University Medical Center, Nashville, TN, USA; 4 Development, Aging and Regeneration Program, Sanford Burnham Prebys Medical Discovery Institute, La Jolla, CA, USA; 5 Department of Medicine, Division of Genetic Medicine & Clinical Pharmacology, Vanderbilt University Medical Center, Nashville, TN, USA; 6 Department of Computer Science, Whiting School of Engineering, Johns Hopkins University, Baltimore, MD 21218, USA; 7 Department of Epidemiology, Harvard T.H. Chan School of Public Health, Boston, MA 02115, USA; 8 Department of Social and Behavioral Sciences, Yale School of Public Health, New Haven, CT, 06520, USA; 9 Environmental Sciences Graduate Program, Oregon State University, Corvalis, OR 97331, USA; 10 Department of Molecular Physiology and Biophysics, Vanderbilt University, Nashville, TN, USA; 11 Department of Internal Medicine, University of Iowa, Iowa City, IA, USA; 12 Department of Pathology, Vanderbilt University Medical Center, Nashville, TN, USA; 13 Department of Cell and Developmental Biology, Vanderbilt University, Nashville, TN, USA; 14 Department of Chemistry, Vanderbilt University, Nashville, TN, USA; 15 Department of Molecular and Human Genetics, Baylor College of Medicine, Houston, TX, USA; 16 Department of Biochemistry and Molecular Biology, East Carolina University, Greenville, NC, USA; 17 Vanderbilt Institute for Global Health, Vanderbilt University Medical Center, Nashville, TN, USA; 18 Department of Pathology, Johns Hopkins University School of Medicine, Baltimore, MD, USA; 19 The Huck Institutes of the Life Sciences; Department of Biochemistry and Molecular Biology, Pennsylvania State University, University Park, PA, USA

**Keywords:** TACO1, Complex IV, Hypertensive heart failure, Renin–angiotensin system, (mRen2)27 transgenic rat, Metabolic remodeling

## Abstract

Mitochondrial dysfunction drives hypertensive heart failure and reflects impaired oxidative phosphorylation and altered organelle structure. The mechanisms linking hypertensive signaling to mitochondrial translation and architecture remain unclear. TACO1 is a mitochondrial translational activator required for cytochrome c oxidase subunit I synthesis and may regulate respiratory chain assembly. We tested whether angiotensin II type 1 receptor activation disrupts TACO1-dependent translation and drives inner membrane remodeling. Using mRen also known as (mRen2)27 hypertensive rat hearts, we assessed mitochondrial function, ultrastructure, and metabolism. AT1R activation reduced TACO1-dependent COX I translation and produced a selective deficiency in complex IV activity. This impaired oxidative phosphorylation and increased the production of reactive oxygen species. Mitochondria exhibited reduced volume, increased fragmentation, and disrupted cristae organization with lower integrity scores. Hypertensive hearts also showed reduced expression of OPA1 and MICOS components. Metabolomic profiling separated control and heart failure groups and revealed enrichment of amino acid, nucleotide, and mitochondrial energy pathways. Lipidomic analysis identified coordinated changes across lipid classes consistent with altered membrane composition. Pharmacological AT1R inhibition restored COX I translation, rescued complex IV activity, and improved cristae structure. These findings establish a mechanistic link between hypertensive signaling, mitochondrial translation, cristae organization, and metabolic remodeling in heart failure.

## 1. Introduction

Cardiovascular disease remains a leading cause of morbidity and mortality worldwide[1]. Hypertensive heart failure reflects a fundamental inability of cardiomyocytes to sustain adenosine triphosphate (ATP) production under conditions of chronic pressure overload[2]. Persistent elevation in afterload increases energetic demand and imposes sustained stress on mitochondrial oxidative metabolism. This continuous demand forces mitochondria to operate near their functional limits, increasing vulnerability to dysfunction. Over time, this imbalance between energy demand and production drives maladaptive remodeling, contractile dysfunction, and progression to heart failure. Despite this progression, the mechanisms linking sustained hypertensive stress to the loss of mitochondrial bioenergetic capacity remain incompletely defined[3].

Hypertensive heart failure associated with mitochondrial dysfunction, which limits ATP production[4]. This impairment reduces electron transport chain activity, diminishes ATP synthesis, and increases production. These defects are often interpreted as metabolic insufficiency. However, they occur alongside reproducible changes in mitochondrial morphology and inner membrane organization[5]. This pattern suggests that impaired respiration arises from coordinated structural remodeling of the organelle rather than an isolated biochemical defect. It remains unclear whether disruption of mitochondrial architecture is sufficient to impair oxidative capacity or whether structural changes arise secondary to declining respiration. Defining this relationship addresses a central gap in understanding the loss of mitochondrial bioenergetic capacity in the hypertensive heart.

In cardiomyocytes, mitochondrial structure regulates respiratory output by affecting cristae architecture and inner membrane organization.[6] Cristae contain the electron transport chain complexes and ATP synthase, making their organization essential for efficient oxidative phosphorylation[5]. Reduced cristae density or loss of defined structure decreases the membrane surface available for electron transport and ATP generation[7]. Fragmentation of the mitochondrial network further disrupts organelle continuity and spatial organization, thereby limiting substrate distribution and reducing metabolic efficiency.[8] In models of hypertensive stress, mitochondria exhibit reduced length, decreased cross-sectional area, and loss of cristae organization. Similar phenotypes are observed following disruption of Optic Atrophy (OPA1), Mitofusin (MFN2), or components of the mitochondrial contact site and cristae organizing system (MICOS), which maintain cristae junctions and inner membrane integrity[9]. These observations support a model in which structural remodeling constrains electron transport efficiency.

Alterations in mitochondrial morphology also influence membrane potential and redox balance.[10] Loss of cristae integrity weakens the proton gradient across the inner membrane, reducing the driving force for ATP synthase activity[11]. Disorganization of respiratory complexes can increase electron leak, leading to elevated superoxide production[12]. These changes reduce the efficiency of oxidative phosphorylation while promoting oxidative stress within the cell. Importantly, these defects can occur in the absence of major reductions in mitochondrial content, indicating that qualitative changes in mitochondrial architecture are sufficient to impair function[6, 13].

These structural defects are reflected in broader metabolic remodeling[14]. In hypertensive and failing hearts, metabolomic profiling demonstrates reductions in tricarboxylic acid cycle intermediates such as citrate and fumarate, along with evidence of impaired fatty acid oxidation[15]. Accumulation of intermediate metabolites and shifts in redox-related pathways indicate reduced flux through oxidative metabolism[16]. These changes are consistent with reduced electron transport efficiency and reinforce the link between mitochondrial structure and metabolic output[17].

Hypertensive stress is therefore associated with a sequence of events that includes mitochondrial structural remodeling, reduced oxidative capacity, and metabolic reprogramming[2, 18]. Disruption of mitochondrial architecture constrains electron transport efficiency, leading to reduced ATP production and increased reactive oxygen species generation[19]. These changes establish a feed-forward cycle that reinforces metabolic dysfunction and contributes to progressive cardiac impairment.

Maintenance of inner membrane organization requires coordinated synthesis and assembly of electron transport chain components. Disruption of mitochondrial protein translation can alter cristae architecture by limiting the availability or proper integration of core respiratory subunits.[20] Translation Accelerator of Cytochrome *c* Oxidase subunit 1-CO1 (TACO1) encodes a mitochondrial translational activator of COX1 that is required for efficient synthesis and assembly of cytochrome c oxidase complex IV[21]. In severe neurodegenerative disorders such as Leigh syndrome, mutations in TACO1 reduce COX1 protein synthesis[22]. This impairs mitochondrial function and contributes to both early and late onset disease. Cardiomyocytes are particularly vulnerable to these defects, as they rely heavily on mitochondrial efficiency and sustained ATP production through oxidative phosphorylation. Changes in TACO1 would critically impact mitochondrial function and contribute to cardiac dysfunction[23]. However, the rational mechanism by which TACO1 modulates mitochondrial complex IV in the presence of candesartan, and how this relates to the complex IV profile in failing human heart has not been previously characterized.

In this study, we test the hypothesis that disruption of mitochondrial architecture is sufficient to impair oxidative capacity under hypertensive stress and that TACO1 contributes to this process by regulating respiratory complex assembly. We examine how defined changes in mitochondrial morphology and cristae organization relate to electron transport efficiency and metabolomic remodeling. This work establishes a mechanistic link between mitochondrial structure and bioenergetic failure and identifies mitochondrial protein translation as a potential.

## 2. Results

### 2.1 Phenome-wide association analysis links *TACO1* loss-of-function to circulatory and endocrine/metabolic phenotypes.

**Figure 1:**
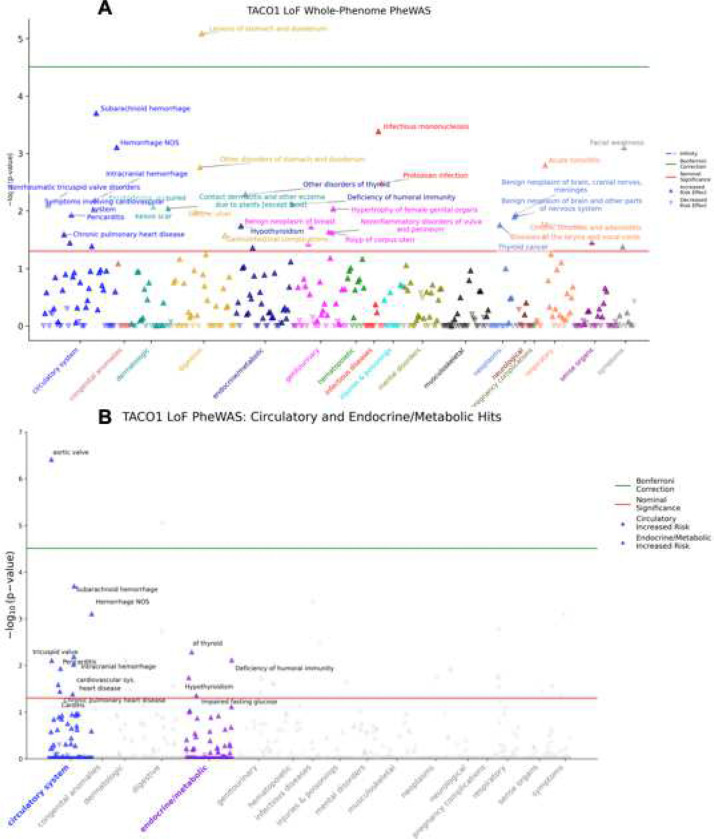
(A) PheWAS Manhattan plot of associations between *TACO1* loss-of-function carrier status and phecode-defined phenotypes in the *All of Us* cohort. Each point corresponds to a single phecode tested in covariate-adjusted logistic regression, with *TACO1* predicted loss-of-function carrier status modeled as the independent variable of interest and phecode case status as the outcome. Models were adjusted for age at last event, sex at birth, and the first five principal components. Phenotypes are arranged by clinical category on the *x*-axis and plotted by −log_10_(*p*-value) on the *y*-axis. Upward triangles denote phenotypes with higher odds in carriers, and downward triangles denote phenotypes with lower odds in carriers. The red line indicates nominal significance (*p* < 0.05), and the green line indicates the Bonferroni correction threshold. (A) Circulatory and endocrine/metabolic phenotypes are highlighted, with nominally significant phenotypes annotated. (B) Whole-phenome PheWAS plot showing the top annotated associations across all phecode categories.

To evaluate whether human genetic variation supports a disease-relevant role for TACO1, we performed a gene-based phenome-wide association study (PheWAS) of TACO1 loss-of-function (LoF) in an EHR-linked cohort (n = 9, LoF carriers = 180,067), testing associations across 1,607 phecodes while adjusting for age, sex at birth, and ancestry principal components. Phenome-wide analysis identified multiple associations spanning several clinical domains (Figure 1A). Notably, several of the strongest signals localized to the circulatory system, including valvular and hemorrhage-related phenotypes, and to endocrine/metabolic phenotypes, including thyroid-related disorders and glycemic traits (Figure 1A–B). The top-ranked circulatory association included nonrheumatic aortic valve disorders (phecode 395.20), which exceeded the Bonferroni significance threshold (OR = 75.26; *p* = 3.87 × 10^−7^) in this analysis (Figure 1B). In addition to that, nominally significant associations clustered among circulatory and endocrine/metabolic phenotypes (Table S1), including subarachnoid hemorrhage (OR = 55.25; *p* = 1.99 × 10^−4^), hemorrhage NOS (OR = 37.49; *p* = 7.83 × 10^−4^), other disorders of thyroid (OR = 10.81; *p* = 5.14 × 10^−3^), intracranial hemorrhage (OR = 18.92; *p* = 6.44 × 10^−3^), nonrheumatic tricuspid valve disorders (OR = 18.25; *p* = 7.86 × 10^−3^), symptoms involving the cardiovascular system (OR = 8.70; *p* = 9.48 × 10^−3^), pericarditis (OR = 15.23; *p* = 1.16 × 10^−2^), hypothyroidism (OR = 5.97; *p* = 1.83 × 10^−2^), chronic pulmonary heart disease (OR = 11.21; *p* = 2.57 × 10^−2^), carditis (OR = 9.70; *p* = 3.59 × 10^−2^), ill-defined descriptions and complications of heart disease (OR = 5.61; *p* = 4.10 × 10^−2^), and impaired fasting glucose (OR = 5.58; *p* = 4.36 × 10^−2^). These findings suggest that reduced TACO1 function is associated with a spectrum of cardiometabolic and vascular phenotypes, providing human genetic support for a potential role for mitochondrial translation in cardiovascular and systemic metabolic regulation.

### 2.2 Laboratory-wide association analysis suggests metabolic alterations associated with TACO1 loss-of-function.

**Figure. 2.**
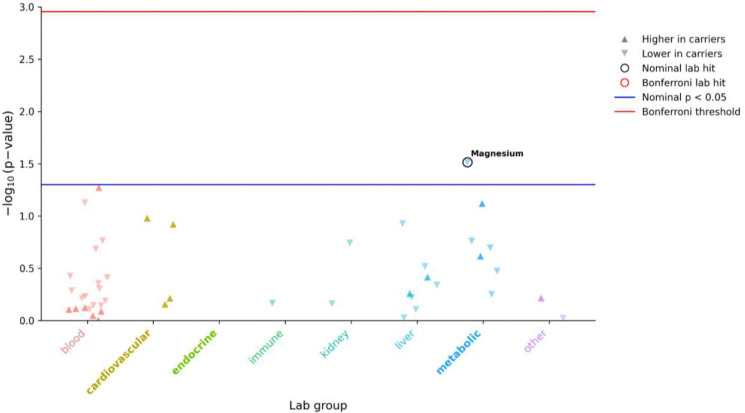
Clinical LabWAS of *TACO1* loss-of-function carrier status in the *All of Us* Research Program. Each point represents a harmonized laboratory phenotype tested using covariate-adjusted linear regression with *TACO1* LoF carrier status as the independent variable. Laboratory values were summarized at the participant level using the median value for each laboratory phenotype, filtered for extreme outliers, and rank-based inverse normal transformed prior to analysis. Laboratory phenotypes are grouped by domain along the *x*-axis, and the *y*-axis represents −log_10_(*p*-value). Upward triangles indicate higher laboratory values in *TACO1* LoF carriers, and downward triangles indicate lower laboratory values in carriers. The blue horizontal line denotes nominal significance (*p* < 0.05), and the red horizontal line denotes the Bonferroni-corrected significance threshold.

To further assess biochemical correlates of TACO1 loss-of-function, we performed a laboratory-wide association study (LabWAS) in the *All of Us* cohort. No laboratory traits reached Bonferroni significance. A nominal association was observed for circulating magnesium levels, which were lower in TACO1 LoF carriers (*β* = −0.64, *p* = 0.031; Figure 2). Magnesium plays a central role in mitochondrial energy metabolism and vascular function, providing biologically consistent support for the role of mitochondrial translation in cardiometabolic regulation.

### 2.3 Hypertensive hemodynamics and indicators of myocardial oxygen consumption in the mRen transgenic model are ameliorated by candesartan treatment.

**Figure 3:**
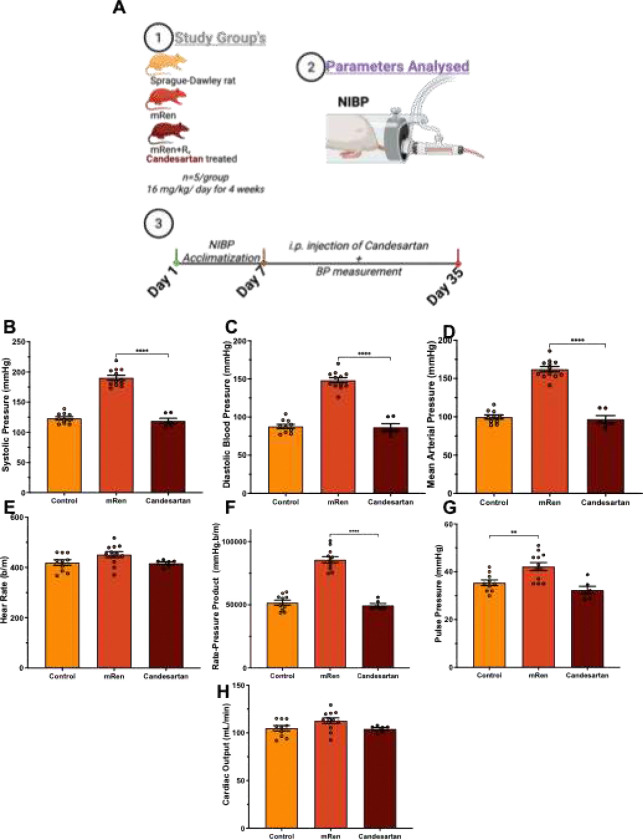
Cardiovascular responses to Angiotensin II sub-type 1 receptor blocker - Candesartan, in(mRen2)27 transgenic hypertension: (A)1–3 is a schematic representation of the study groups, methodology involved, and treatment methodology. of the Cardiovascular responses to Angiotensin II sub-type 1 receptor blocker - Candesartan, in mRen2 transgenic model of hypertension: Systolic (B), Diastolic (C), Mean Arterial Pressure (D), calculated indicator of myocardial oxygen consumption – Rate Pressure Product (F), Pulse Pressure (G), were significantly higher in (mRen2)27 vs control. Values are mean SEM (**** p<0.0001, ** p<0.01). Candesartan restored all responses to control levels. However, no changes in cardiac output (H) and the Heart Rate (E) were observed.

Non-invasive tail-cuff plethysmography measurement (Figure 3A) demonstrated that transgenic mRen rats exhibited significantly elevated blood pressure (BP) compared with normotensive SD controls, with no difference in heart rate (HR) and cardiac output (CO). Cardiovascular parameters were measured daily, and 35 days of treatment with candesartan (also described as mRen+R_x_) (16 mg/kg body weight) normalized systolic blood pressure (SBP), diastolic blood pressure (DBP), pulse pressure (PP), mean arterial pressure (MAP), and rate-pressure product (RPP); an index of myocardial oxygen demand, but there were no changes in the cardiac output (CO) and heart rate in mRen rats, restoring these parameters to levels comparable to controls.

### 2.4 Left Ventricle proteomics identifies TACO1 as a candesartan-responsive mitochondrial protein in chronic RAAS overactivation.

**Figure 4:**
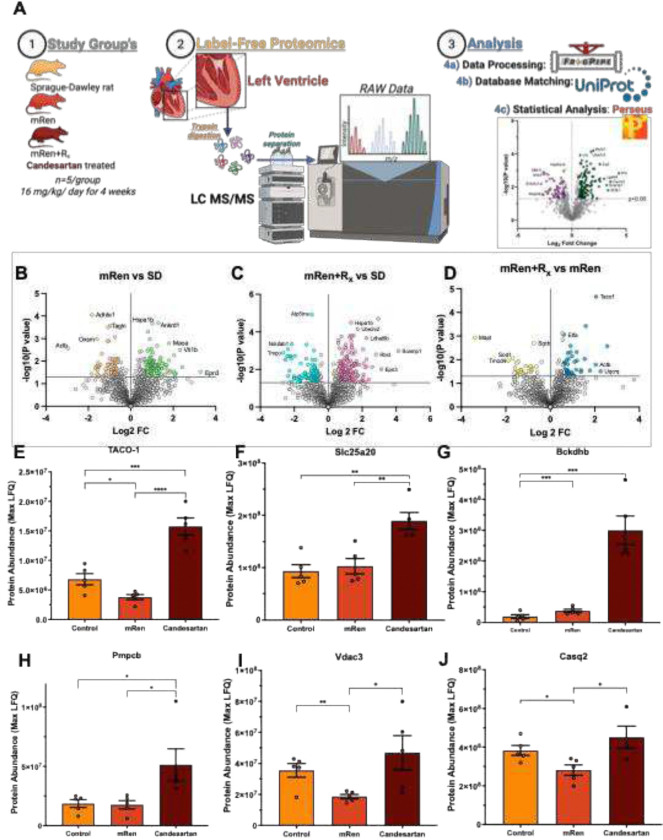
(A) A schematic representation of comparative proteomic workflow of left ventricular tissue (1) Study Groups: Sprague-Dawley (SD), mRen, and treated mRen (mRen+R_x_) rat models. (2) Label-Free Proteomics workflow and (3) Data processing and Bioinformatics analysis. (B-D) Volcano plots of differentially expressed protein based on their statistical significance (−log_10_ P-value) and fold changes (log_2_) (E-J): Effects of Candesartan on Mitochondrial Protein Import Assembly, Antioxidant-Related proteins and calcium handling in LV heart: TACO1, Pmpcb, Slc25a20, Bckdhb, Vdac3 and Casq2 genes: Proteomic Analysis indicated upregulation in the presence of candesartan Cilexetil. Vdac3 and Casq2 genes provide instructions for storing and transporting calcium in the heart muscle cells. The protein is a major calcium reservoir in the sarcoplasmic reticulum and helps regulate the release of calcium ions through the RYR2 channel to control heart muscle contractions.

To obtain an unbiased, system-level characterization of the left ventricular proteome across experimental groups and to identify candidate proteins involved in RAAS-induced mitochondrial dysfunction, label-free quantitative proteomic analysis was performed. To support the volcano plot results, Figure 4 (B–D) shows log_2_ abundance and relative abundance distributions for the highlighted proteins across biological replicates, demonstrating that the group differences reflect coherent shifts rather than outlier-driven effects.

Left ventricular tissue was collected from SD controls, untreated mRen rats, and mRen+R_x_ (n = 5/group). Samples were subjected to tryptic digestion, and the resulting peptides were separated and analyzed using LC–MS/MS. Raw data were processed with FragPipe and statistically evaluated using Perseus, enabling protein identification and quantification against the UniProt database (Figure 4A). Compared with the control group, the hypertensive group exhibited widespread proteomic alterations, including numerous significantly up- and downregulated proteins, with several high-confidence candidates linked to cardiac metabolism and mitochondrial function. In contrast, the proteome of candesartan-treated mRen animals displayed a distinct expression profile relative to controls, showing only partial overlap with that of the untreated mRen hypertensive group. This pattern suggests that AT1R blockade induces a proteomic shift that is not simply a reversal of RAAS-driven changes. Notably, a direct comparison between the mRen+R_x_ and untreated mRen groups identified TACO1 as the most significantly upregulated protein, appearing as the top hit on the volcano plot (Figure 4B, -2C, and 2D), consistent with its suppression under chronic RAAS activation and its restoration following AT1R blockade [43, 44].

TACO1 (Figure 4E) levels were significantly reduced in left ventricular tissue from untreated mRen rats compared with controls. In contrast, mRen+R_x_ treatment markedly increased TACO1 abundance, exceeding levels observed in both untreated mRen and control groups (p<0.0001). Targeted quantification confirmed that the volcano plot reflects coordinated proteomic remodeling of mitochondrial metabolism, organelle maintenance, and calcium handling in the hypertensive left ventricle. Bckdhb and Slc25a20 (Figure 4F&4G) coordinates to branched-chain amino acid catabolism and fatty-acid import for β-oxidation exhibited a recovery in the presence of candesartan. These results indicate bidirectional, pharmacologically reversible regulation of TACO1 via AT1R signaling in the hypertensive heart. In parallel, Peptidase, Mitochondrial Processing Beta Subunit (Pmpcb) increased with candesartan (p<0.05; Figure 4H), consistent with enhanced mitochondrial protein processing capacity. In contrast, the cardiac calcium-handling protein Casq2 (Figure 4J) was significantly reduced in the left ventricular tissue of untreated mRen rats compared with controls indicating impaired sarcoplasmic reticulum calcium storage in the hypertensive state. Collectively, these findings identify TACO1 as a candesartan-responsive regulator of mitochondrial translation, whose suppression during chronic RAAS overactivation and restoration with AT1R blockade represents a key feature of left ventricular proteome remodeling in hypertensive cardiomyopathy.

### 2.5 Angiotensin receptor subtypes are expressed in excitable tissues

To understand the chronic hemodynamic stress underlying hypertension and mitochondrial changes observed in excitable tissue and to confirm the localization of angiotensin-mediated receptors, the mRen transgenic rat model, linked to chronic RAAS activation mechanistically to mitochondrial remodeling was studied. mRen rodent is a monogenic model generated by stable integration of the murine Ren-2d renin gene that produces severe, Angiotensin II–dependent hypertension with prominent extrarenal RAAS activity and strong responsiveness to AT1 receptor blockade [41, 42]. To demonstrate constitutive RAAS activation in the mRen transgenic rat the presence of RAAS receptors in excitable sympathetic nerve of superior cervical ganglion (SCG) were confirmed by immunofluorescence in both SD control and mRen groups.

**Figure 5:**
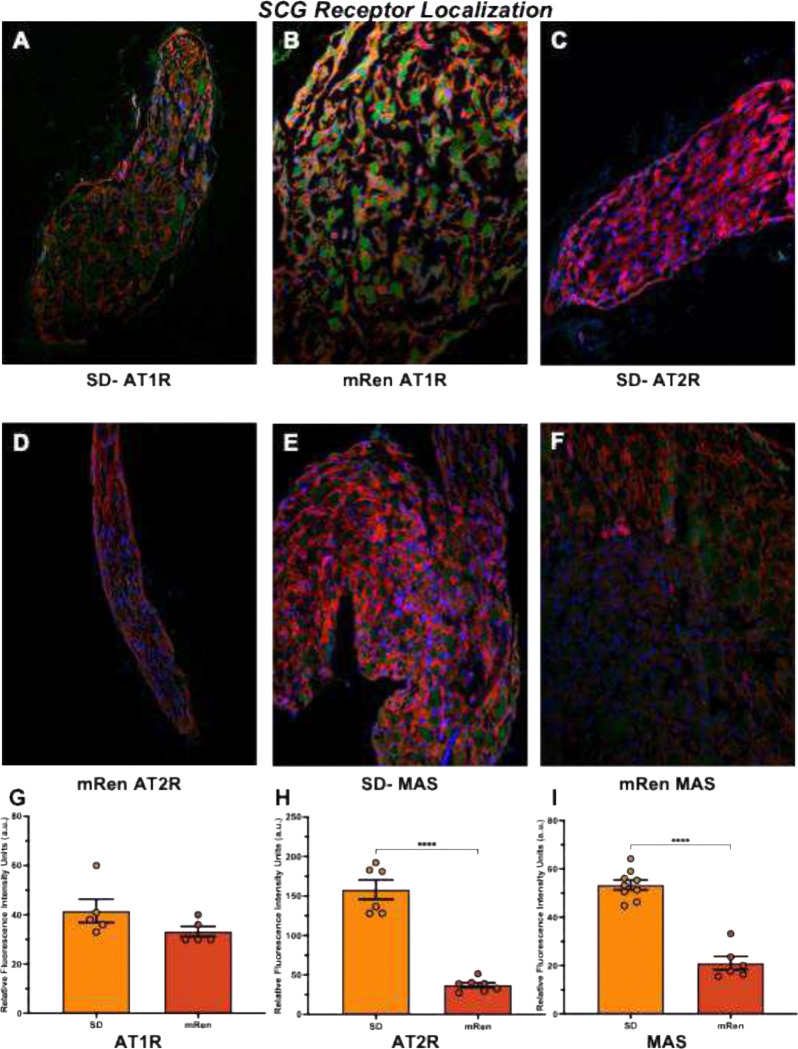
Confocal microscopy image of the Superior Cervical Ganglia from mRen2–27 Rat and their fluorescent targets in SD and mRen. (A-B) Antibody targets AT1—extracellular-ATTO Fluor-550; (C-D) AT2—extracellular-ATTO Fluor-488 and (E-F) MAS receptor –Fluor-488 tags (1° antibody dilution 1:50), 4′, 6-diamidino-2-phenylindole and F-actin merged images show the green fluorescence is specific for RAAS receptors. (G-H) Image analysis performed by ImageJ software and relative fluorescence intensity units are presented values in each panel are mean ± SEM; *P < 0.05.

The fluorescent targets for AT1, AT2, and MAS-Oncogene receptors are visualized by confocal microscopy (Fig. 5). The 4’, 6-diamidino-2-phenylindole and F-actin merged images show the green fluorescence intensity specific for angiotensin receptors (Figs. 5A–F). There are no differences between strains in the relative fluorescence intensity units for AT1 and MAS receptors, but a significantly lower signal was detected in the immunofluorescence images for AT2 receptors (188±4; n = 4 vs. 44 ± 5 a.u. n = 6; ***P<0.0001). From the hypertensive rats, consistent with the lower receptor protein and mRNA expression in mRen compared with age matched control SCG. AngII generally acts through binding to AT1 and AT2 G-protein-coupled receptors (GPCR), the effects of AT1 receptors mediate excitatory responses, and AT2 serves as the protective arm of RAAS at the sympathetic ganglia. Angiotensin-converting enzyme-2 (ACE2) converts AngII into Ang (1–7) mediated by MAS receptor, a GPCR for Ang-(1–7), but not for AngII and it is also inhibitory in response. Consequently, an increase in excitatory AT1 receptors may be expected to facilitate sympathetic outflow. However, a decrease in receptor expression for AT2 and MAS would tend to enhance the effect on the synaptic transmission, with or without elevated levels of AT1 receptors [37].

### 2.6 Mitochondrial population is disorganized in Ventricular Heart Failure

**Figure 6:**
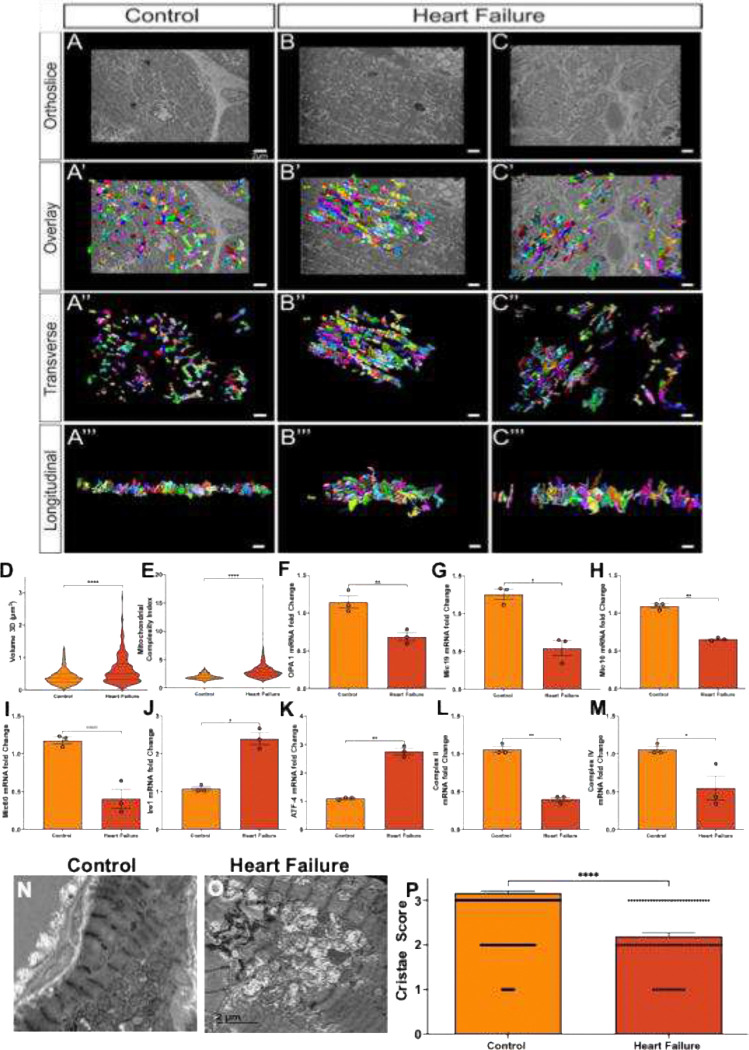
The Representative serial block-face scanning electron microscopy (SBF-SEM) of ortho slices from myocardial volumes in Control (A) and Heart Failure (B and C), The same Ortho slices with mitochondrial segmentations overlaid; individual 3D reconstruction of mitochondria are colored to visualize object boundaries and spatial distribution (A’’ to C″). Transverse views of 3D mitochondrial reconstructions from the corresponding SBF-SEM volumes (A”’ to C”’). Longitudinal views of the reconstructed mitochondria highlighting changes in mitochondrial organization between control and heart failure. Scale bars, 2 μm.

Serial block-face scanning electron microscopy (SBF-SEM) revealed the three-dimensional organization of cardiac mitochondria. In control cardiomyocytes (Figure 6A–A”’), mitochondria formed densely interconnected, parallel columns aligned with myofibrils, consistent with an orderly and highly functional network. In contrast, heart failure cardiomyocytes (Figure 6B–C and B”’–C”’) showed marked disorganization, with smaller, fragmented mitochondria dispersed irregularly throughout the cytoplasm and loss of the longitudinal network structure. Quantitative 3D confocal analysis demonstrated a significant increase in mitochondrial volume and network measures in heart failure (p<0.0001; Figure 6D). Morphological analysis similarly showed a significant increase in mitochondrial complexity relative to volume (p<0.0001; Figure 6E), indicating a shift toward a fragmented and simplified mitochondrial phenotype.

To examine inner mitochondrial membrane remodeling, qRT-PCR was performed for key regulators of mitochondrial structure and stress responses, including Optic Atrophy 1 (OPA1), mitochondrial contact site and cristae organizing system (MICOS) subunits (Mic10, Mic19, Mic60), and stress markers Inositol-Requiring Enzyme 1 (IRE1) and Activating Transcription Factor 4 (ATF4), along with components of the respiratory chain. OPA1 expression was significantly reduced in heart failure compared to control (p<0.05; Figure 6E). Among MICOS subunits, Mic10 and Mic60 were also significantly decreased (p<0.001 and p<0.01; Figures 4G and 4H), while Mic19 showed a non-significant reduction (Figure 4F). These changes suggest disruption of the structural machinery that maintains cristae organization and inner membrane integrity. Markers of mitochondrial stress were elevated in heart failure tissue, with significant upregulation of IRE1 (p<0.01) and ATF4 (p<0.001; Figures 6I and 6J), indicating activation of proteostatic stress pathways. At the same time, transcript levels of respiratory complex components were reduced, with significant decreases in Complex II (p<0.05) and Complex IV (p< 0.001; Figures 6K and 6L). Together, these findings link mitochondrial structural disruption with impaired respiratory capacity and activation of stress responses in heart failure.

### 2.7 Metabolomics and lipidomics

**Figure 7:**
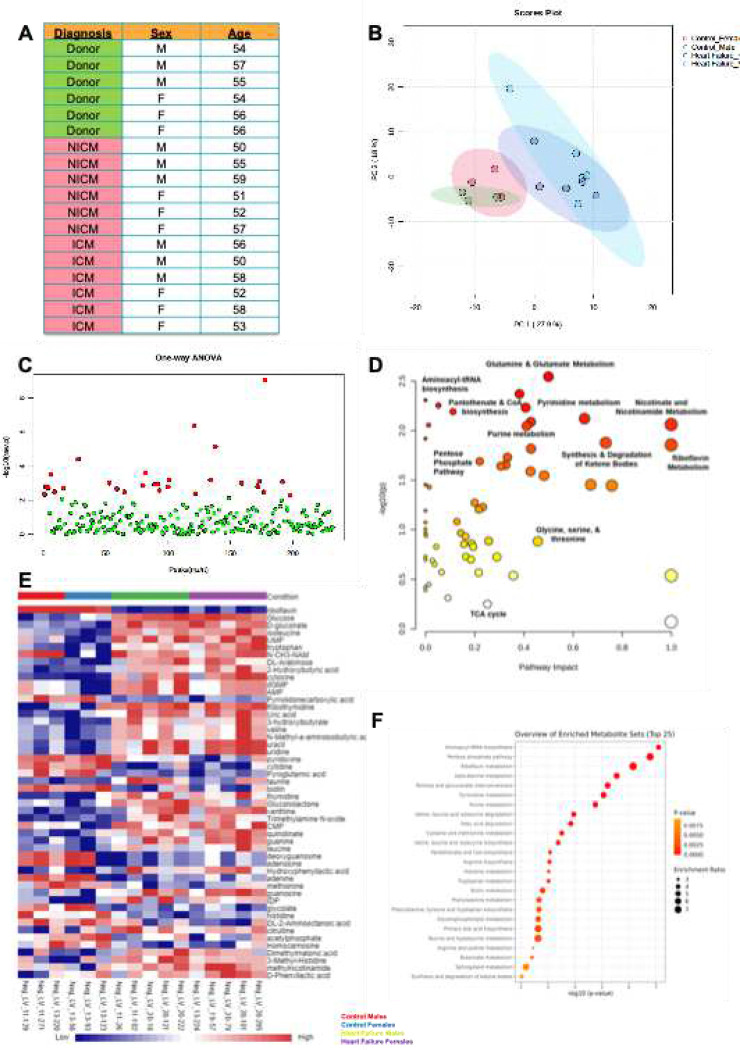
Metabolomic remodeling of the normal and heart failure left ventricle. A) Donor control, non-ischemic cardiomyopathy (NICM), and ischemic cardiomyopathy (ICM) samples with identifiers including diagnosis, sex, and age. B) Principal Component analysis score of metabolic profile describing control and heart failure. C) Illustration demonstrating metabolites that are significantly altered in the experimental groups. Points in the illustration represent individual metabolites plotted against mass-to-charge ratio (m/z). D) Analysis of metabolic pathways that are significantly altered, highlighting enrichment of the pentose phosphate pathway, and oxidative metabolism–associated pathways. Pathway impact scores reflect relative pathway modifications that deviate from normal physiological conditions. E) Heatmap of metabolites across sex and sample group, with relative metabolite abundance represented by blue for low and red for high. F) Top 25 metabolites based on enrichment ratio and statistically significant in heart failure.

Principal Component Analysis (Figure 7B) was conducted to compare control samples with those from heart failure patients along the principal component axes. When comparing the two groups, we observed more extensive metabolic reprogramming in the heart failure group. Regardless of gender, the metabolic profiles remained consistent within each group. A subset of metabolites that were altered was identified by one-way ANOVA (Figure 7C). Pathway impact results, (Figure 7D), indicate coordinated remodeling of biosynthetic and redox-balancing pathways in heart failure subjects. Heatmap visualization revealed consistent clustering of heart failure samples distinct from controls, with coordinated upregulation and downregulation of metabolite sets across disease states (Figure 7E). This pattern supports the presence of a conserved metabolic remodeling program associated with cardiac dysfunction. (Figure 7F) suggests a significant enrichment of amino acid metabolism (including glutamine, glycine, serine, and threonine pathways), nucleotide biosynthesis/degradation, pentose phosphate pathway activity, and oxidative metabolism-related pathways.

**Figure 8:**
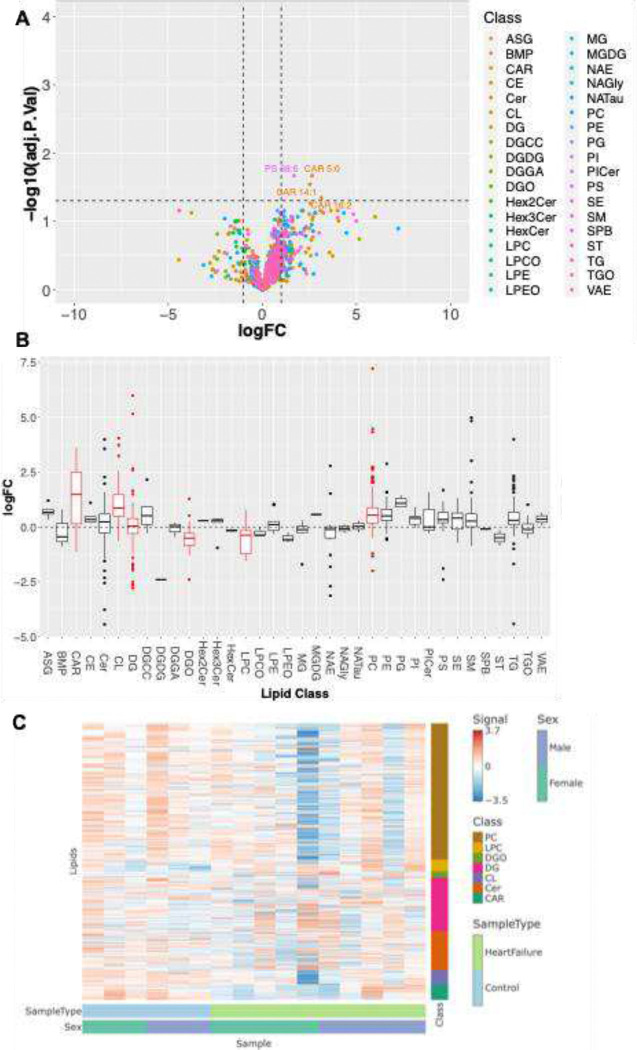
Heart failure is associated with class-specific lipidomic remodeling. (A) Volcano plot showing differential lipid abundance between heart failure and control myocardium. Each point represents an individual lipid species, colored by lipid class. The x-axis denotes log_2_ fold change (log_FC_), and the y-axis denotes −log_10_ adjusted P value. Dashed vertical lines indicate fold-change thresholds, and the dashed horizontal line denotes the significance cutoff. Select significantly altered lipid species are annotated. (B) Box-and-whisker plots summarizing log_2_ fold change distributions across lipid classes. Red outlines indicate lipid classes with statistically significant changes, whereas black outlines indicate non-significant classes. (C) Heatmap of differentially abundant lipid species across individual samples. Lipid abundances are Z-score normalized and hierarchically clustered. Sample annotations

In comparison to control myocardium (Figure 8A), the differential lipid analysis of a class of lipid species was significantly altered in heart failure. Most of the lipids were low; a distinctive modulation was observed, indicating a selective remodeling rather than global lipid alteration. Lipid changes were examined at the class level (Figure 8B). Many lipid classes exhibited non-specific alterations in lipid metabolic pathways rather than pivoting toward a specific lipid species. However, we observed six classes of lipids, including acylcarnitines (CAR), cardiolipin) (CL), diacylglycerol (DG), diacylglycerol-related species) (DGO), lysophosphatidylcholine) (LPC), and phosphatidylcholine (PC) increased in the heart failure case, attributed to a targeted remodeling of lipids. The hierarchical clustering pathways (Figure 8C) related to PC, LPC, DGO, DG, CL, Car, and CAR in control and heart failure cases. The lipids associated with mitochondrial membrane-CL, phospholipid turnover -PC, PCL, mitochondrial fatty acid handling-CAR, and lipid signaling intermediates (DG/DGO) in heart failure reveal the presence of a conserved lipid remodeling program associated with cardiac dysfunction, which is specific to the heart regardless of sex.

### 2.8 Structural and Functional Remodeling in siTACO1 hiPSC-CMs

**Figure 9.**
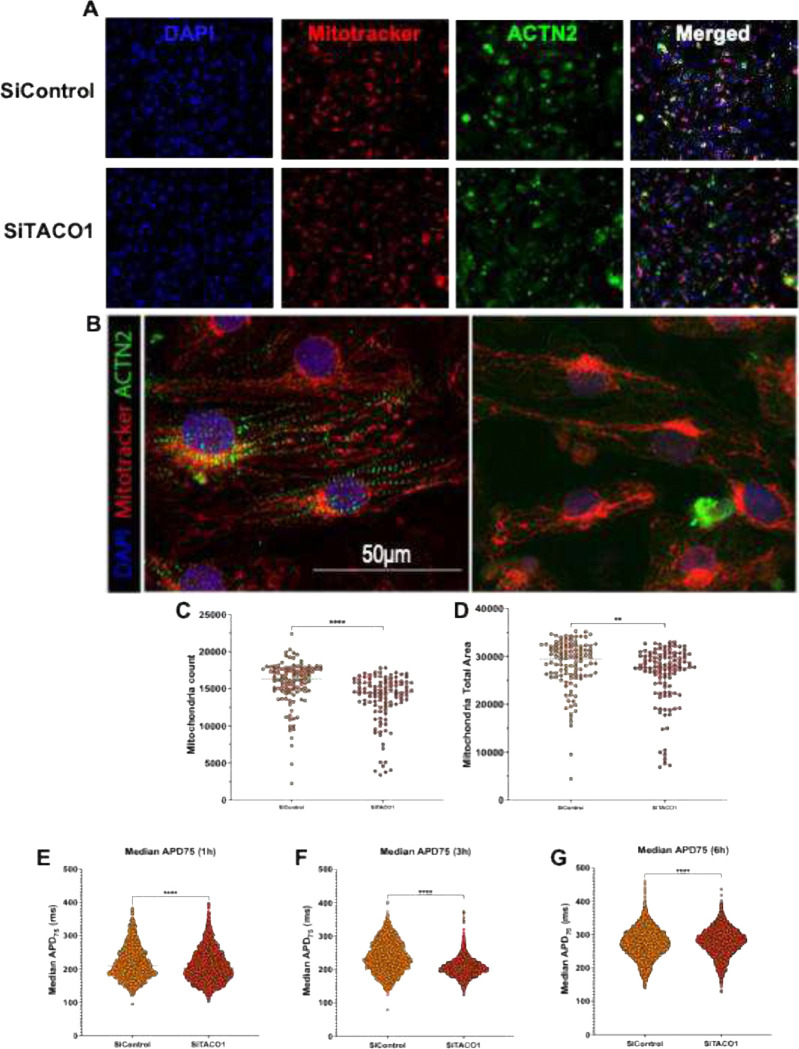
TACO1 Silencing Reshapes Mitochondrial Architecture and Alters Electrophysiological Properties of Ventricular-Like hiPSCs. (A) Representative low-magnification fluorescence image of hiPSCs transfected with SiControl or SiTACO1, nuclei stained with DAPI, mitochondria MitoTracker, and sarcomeric α-actinin ACTN2 with merged channels shown. (B) High-magnification microscopy images highlighting mitochondrial distribution in siControl and siTACO1 hiPSCs. Mitochondrial quantification (C) Mitochondrial Count and (D) Total mitochondrial area. (E-G) Population distributions of median APD75 (ms) at (E) 1 h, (F) 3 h, and (G) 6 h in siControl versus siTACO1 groups, derived from optical voltage recordings. APD75 distributions were analyzed at single-cell resolution using a population-based framework to capture heterogeneity across cells.

**Figure 10.**
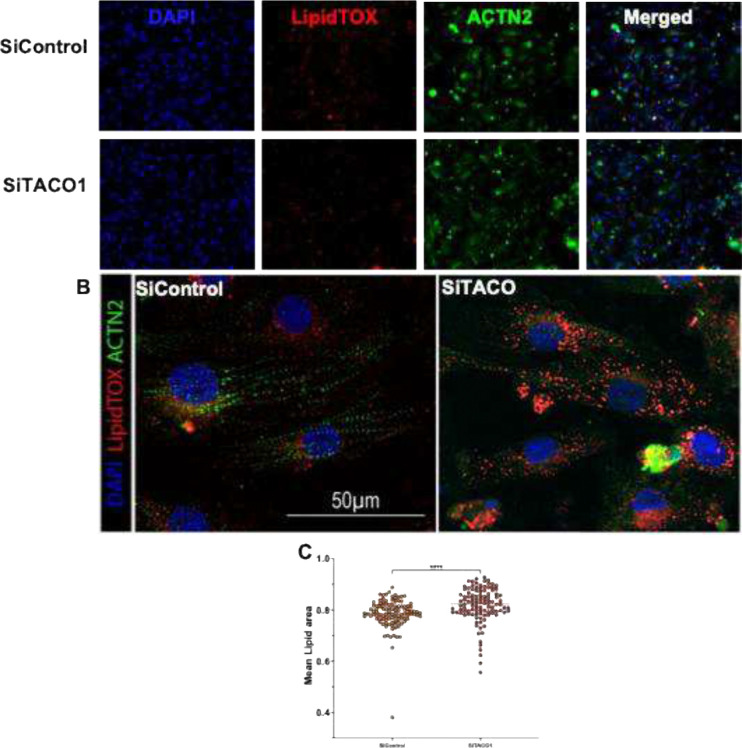
TACO1 Silencing Reshapes Mitochondrial Architecture and Alters Electrophysiological Properties of Ventricular-Like hiPSCs. (A) Representative low-magnification fluorescence image of hiPSCs transfected with SiControl or SiTACO1, nuclei stained with DAPI, neutral lipid droplets stained with LipidTOX, and sarcomeric α-actinin ACTN2 with merged channels shown. (B) Lipid distribution relative to sarcomere structure in siControl and siTACO1 cardiomyocytes in high magnification. (C) Quantification of mean lipid area per condition derived from automated image segmentation and feature extraction.

Morphological analysis (Figure 9A–B) of TACO1 knockdown on mitochondrial remodeling in hiPSC-CMs derived cardiomyocytes revealed mitochondrial changes (segmentation quality control shown in Figure S3). Significant reduction in mitochondrial count (Figure 9C) and a decrease in total mitochondrial area (Figure 9C) in the siTACO1 condition relative to siControl. On the other hand, the cells electrophysiological outcomes measured in action potential duration at 75% repolarization (Figure 9E–G) showed that siTACO1 cardiomyocytes exhibited significant shifts in APD75 distributions compared with controls across the time course (1 h, 3 h, and 6 h). This implies that silencing TACO1 resulted in altered repolarization dynamics and increased heterogeneity at the single-cell population level. Lipid accumulation in hiPSC-CMs imaged (Figure 10A–B) using LipidTOX demonstrated an increase in mean lipid area (Figure 10C) in the TACO1 silenced group in comparison to Si Control indicating alteration in lipid handling and metabolic remodeling at a cellular level.

### 2.9 TACO1 knockdown recapitulates OPA1- and MFN2-deficient mitochondrial ultrastructural and bioenergetic defects, impairing oxidative phosphorylation in cardiomyocytes.

**Figure 11.**
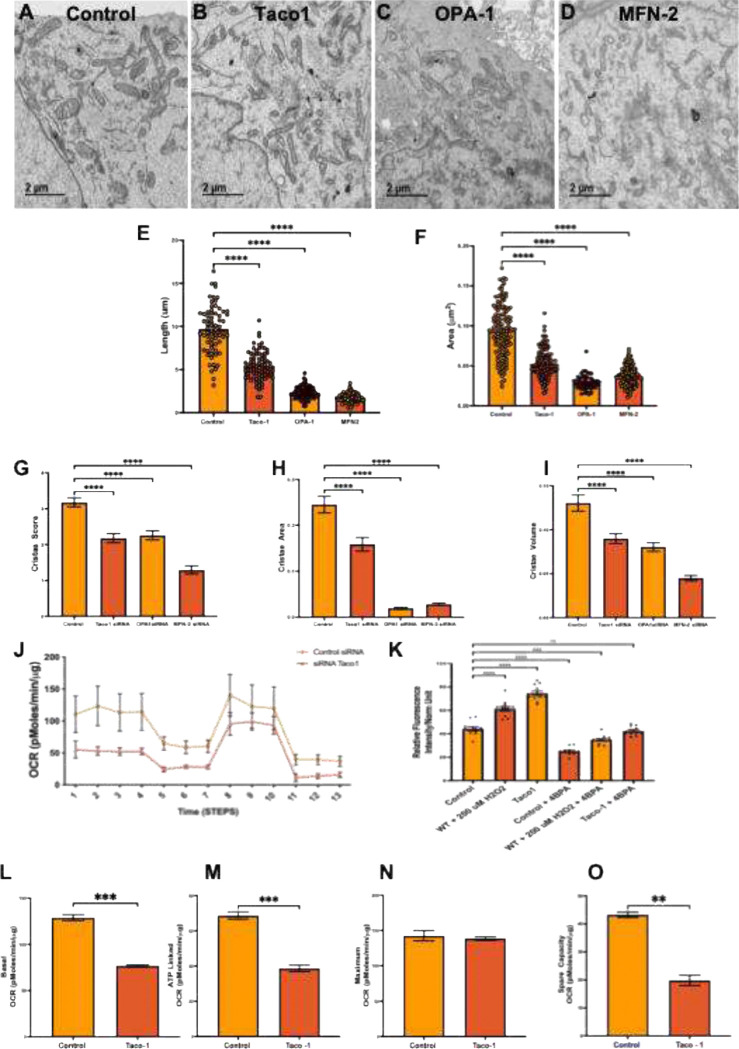
TACO1 knockdown disrupts mitochondrial morphology, cristae ultrastructure, and respiratory function. (A–D) Representative transmission electron microscopy (TEM) images of mitochondria in cells treated with control (A), Taco1 siRNA (B), OPA-1 siRNA (C), or MFN-2 siRNA (D). Scale bars = 2 μm.(E–F) Quantification of mitochondrial length (E) and cross-sectional area (F) from TEM images across control, Taco-1, OPA-1, and MFN-2 knockdown conditions. Individual data points are overlaid on bar graphs; horizontal brackets indicate statistically significant pairwise comparisons. (G–I) Quantification of cristae score (G), cristae area (H), and cristae volume (I) in cells transfected with control, Taco1, OPA1, or MFN-2 siRNA. Data represent mean ± SEM.(J) Oxygen consumption rate (OCR) measured by Seahorse XF assay over sequential measurement steps in cells transfected with control siRNA (gold) or Taco1 siRNA (orange). The step protocol implies injections of mitochondrial stress test compounds.(K) Relative mitochondrial membrane potential (fluorescence intensity normalized to cell area) in control, Taco1 knockdown, and cells treated with 200 μM H_2_O_2_ and/or the mitochondrial protectant 4-BPA, as indicated.(L–O) Quantification of basal OCR (L), ATP-linked OCR (M), maximum OCR (N), and spare respiratory capacity (O) in control and Taco-1 knockdown cells derived from the Seahorse mito stress test. All graphs show mean ± SEM. Statistical significance determined by one-way ANOVA with post-hoc multiple comparisons or unpaired t-test, as appropriate. **p < 0.01, ***p < 0.001, ****p < 0.0001; ns, not significant.

To define the functional consequences of TACO1 depletion on mitochondrial ultrastructure and to compare these effects with those of OPA1 and MFN2 deficiency, transmission electron microscopy was performed on cardiomyocytes transfected with siRNA targeting TACO1, OPA1, or MFN2, alongside control siRNA. As shown in the representative micrographs (Figure 11A), control cardiomyocytes exhibited normal ultrastructure, characterized by appropriately sized and elongated mitochondria with well-organized lamellar cristae, consistent with a metabolically active phenotype. In contrast, TACO1 knockdown resulted in marked ultrastructural abnormalities, including smaller, more rounded mitochondria and disrupted inner membrane organization, indicative of impaired mitochondrial integrity. Comparable mitochondrial ultrastructural abnormalities were observed following OPA1 knockdown, characterized by fragmented mitochondria and disrupted cristae organization (Figure 11A, OPA1), as well as after MFN2 knockdown, where mitochondria also exhibited pronounced structural disruption and fragmentation.

These observations were quantitatively validated by morphometric analysis. Cristae volume was significantly reduced in cardiomyocytes with TACO1, OPA1, or MFN2 knockdown compared with siRNA controls (Figure 9B; p < 0.0001 for all groups), indicating that loss of TACO1 impairs cristae architecture to a degree comparable to disruption of established mitochondrial dynamics regulators. Consistently, mitochondrial length was markedly decreased across all knockdown conditions relative to controls (Figure 11E; p < 0.0001), reflecting a shift toward a fragmented mitochondrial phenotype. Similarly, mitochondrial cross-sectional was reduced in TACO1-, OPA1, and MFN2-deficient cells (Figure 11D), reaching statistical significance in TACO1 and OPA1 knockdown (p < 0.0001) and in MFN2 knockdown (p < 0.001). In addition, the cristae score, a composite metric of inner membrane organization, was significantly decreased in all knockdown groups compared with controls, further supporting a shared disruption of mitochondrial ultrastructural integrity.

To determine whether the ultrastructural defects induced by TACO1 knockdown are accompanied by impaired mitochondrial bioenergetics, oxygen consumption rate (OCR) was assessed using Seahorse XF extracellular flux analysis in cardiomyocytes transfected with control or TACO1 siRNA. As shown in the OCR traces following sequential inhibitor injections (Figure 92I), TACO1-deficient cells exhibited a marked reduction in respiratory capacity across all phases of the assay compared with controls. Basal respiration was significantly decreased in TACO1-knockdown cardiomyocytes (Figure 11G), and maximal respiration following FCCP treatment showed a reduction trend relative to controls (Figure 11H), indicating impaired electron transport chain capacity. Consistently, spare respiratory capacity was significantly diminished (Figure 11K), as was ATP-linked respiration (Figure 11M), reflecting reduced mitochondrial ATP-generating efficiency. Validation of siRNA-mediated knockdown confirmed effective suppression of target proteins across experimental conditions, and combined knockdown of TACO1 with mitochondrial dynamics regulators (OPA1 and MFN2) produced additive effects on mitochondrial structure (Figure 11J).

### 2.10 TACO1 Knockdown Disrupts Mitochondrial Morphology, Cristae Ultrastructure, and Respiratory Function.

To investigate the role of TACO1 in maintaining mitochondrial integrity, we depleted TACO1 by siRNA-mediated knockdown and compared the resulting phenotypes to those induced by loss of OPA1 and MFN2. TEM analysis revealed that TACO1 depletion significantly reduced mitochondrial length and cross-sectional area relative to controls (Figure 11A–F; p < 0.0001), phenocopying the fragmentation observed upon OPA1 and MFN2 knockdown. TACO1 loss also significantly impaired cristae architecture, with reductions in cristae score, area, and volume comparable to those seen with OPA1 and MFN2 depletion (Figure 11G–I; p < 0.0001). Consistent with these structural defects, TACO1 knockdown cells exhibited significantly reduced mitochondrial membrane potential under basal conditions, an effect that was partially rescued by treatment with the mitochondrial protectant 4-BPA (Figure 9K; p < 0.0001). Seahorse XF Mito Stress Test profiling further revealed that TACO1 depletion significantly impaired basal OCR and ATP-linked OCR (Figure 11J–M; p < 0.001) and markedly reduced spare respiratory capacity (Figure 11O; p < 0.01), while maximal OCR was not significantly affected (Figure 11N). Collectively, these data establish TACO1 as a regulator of mitochondrial morphology, cristae integrity, and bioenergetic function, positioning it as a previously underappreciated contributor to mitochondrial homeostasis.

### 2.11 TACO1 Loss Reduces Mitochondrial Size and Increases Sphericity Across Multiple Cell Lines

To further characterize the impact of TACO1 loss on mitochondrial morphology in three dimensions, we performed 3D volumetric reconstruction of the mitochondrial network in both HEK and HeLa cells and quantified mitochondrial area, volume, and sphericity. In HEK cells, DMSO-treated controls displayed an elaborately interconnected mitochondrial network with large, elongated mitochondria (Figure S4A). In contrast, treatment with Mysl22, Mfi8, MiclXin, or TACO1 each resulted in a visually apparent fragmentation and condensation of the network (Figure S4B–E). Quantitative analysis confirmed that mitochondrial area and volume were significantly reduced across all treatment conditions relative to DMSO-treated controls (Figure S3F–G; p < 0.0001 for all comparisons). Concordantly, mitochondrial sphericity was significantly increased in all treatment groups (Figure S4H; p < 0.0001), reflecting a shift toward rounder, more fragmented mitochondria. Consistent findings were observed in HeLa cells. Control siRNA-transfected cells exhibited an expansive, networked mitochondrial morphology (Figure S4I), which was markedly disrupted by knockdown of MiCOS, OPA1, MFN2, or TACO1, with mitochondria appearing smaller and more punctate across all knockdown conditions (Figure S4J–M). Quantification confirmed significant reductions in both mitochondrial area and volume in all knockdown groups relative to controls (Figure S4O–P; p < 0.0001 for all comparisons). Sphericity was similarly and significantly increased across all -knockdown conditions (Figure S4Q; p < 0.0001), further corroborating a shift toward fragmented mitochondrial morphology. Together, these 3D morphometric analyses demonstrate that TACO1 depletion robustly reduces mitochondrial size and promotes sphericity across multiple cell types, reinforcing its role as a regulator of mitochondrial network integrity alongside established morphology regulators including MiCOS, OPA1, and MFN2.

### 2.12 TACO1 Knockdown Drives Peripheral Redistribution of Mitochondria in HeLA Cells

To determine whether TACO1 loss affects the spatial organization of the mitochondrial network, we performed MitoTracker-based fluorescence imaging in HeLa cells transfected with siRNAs targeting TACO1, MICOS, OPA1, or MFN2, and quantified mitochondrial distribution using a concentric-circle analysis framework. In control cells, MitoTracker fluorescence was distributed broadly across the cytoplasm, a pattern that was not significantly altered by knockdown of MICOS, OPA1, or MFN2 (Figure S5A–B). By contrast, TACO1 depletion drove a striking and selective redistribution of the mitochondrial network toward the cell periphery, with fluorescence intensity in the radial and distal zones significantly elevated compared to all other conditions (Figure S4B; p < 0.001 and p < 0.0001, respectively). Notably, distal zone (Figure S5) fluorescence in TACO1 knockdown cells reached approximately 75% of total cellular signal, nearly double that observed in controls. These findings reveal a specific role for TACO1 in governing intracellular mitochondrial positioning that is not shared by other mitochondrial morphology regulators and may reflect disrupted interactions between the mitochondrial network and cytoskeletal transport machinery.

## 3. Discussion

In this study, we identify TACO1 as a key regulator coupling mitochondrial protein translation to cristae architecture and respiratory chain efficiency in the setting of hypertensive heart failure. By incorporating human genetics, ultrastructural, molecular, metabolic, and physiological analyses, our findings position mitochondrial translation as an important control point through which neurohormonal stress impacts mitochondrial organization and bioenergetic capacity in the heart.

We first anchored this framework in human population data by asking whether rare predicted TACO1 loss-of-function was associated with clinical phenotypes or laboratory traits relevant to cardiometabolic disease. The presence of a Bonferroni-significant association with aortic valve disorders aligned with the central role of TACO1 impairment. This suggests that translation-linked respiratory chain constraints may contribute to structural cardiovascular vulnerability. The additional clustering of hemorrhage-related phecodes is directionally consistent with a broader vascular phenotype and merits follow-up in independent cohorts. We also identified laboratory traits linking lower circulating magnesium in TACO1 LoF carriers, which is biologically consistent with altered mitochondrial energetics, vascular regulation, and cardiometabolic stress. These human genetic and laboratory-based associations provide clinical context implicating reduced TACO1 function in cardiometabolic vulnerability.

Loss of TACO1 impairs mitochondrial translation and compromises complex IV function leading to defects in oxidative phosphorylation and cardiac dysfunction in experimental models. Ultrastructural analyses across human samples and disease models consistently revealed mitochondrial fragmentation, reduced organelle size, and loss of cristae integrity. These morphological changes were accompanied by reduced expression of OPA1 and multiple components of the MICOS complex, supporting coordinated disruption of inner mitochondrial membrane organization. Together, these data indicate that impaired mitochondrial translation is closely linked to remodeling cristae architecture under pathological stress. The observed transcriptional remodeling of OPA1 and MICOS subunits is consistent with patterns reported in aging and stressed mitochondria and suggests engagement of adaptive programs that regulate membrane structure in response to bioenergetic challenge. However, whether these transcriptional changes are causative or represent secondary responses to impaired respiratory chain function remains unresolved. The convergence of structural and transcriptional alterations nevertheless highlights inner membrane organization as a sensitive target of disease associated with mitochondrial stress.

From a biophysical perspective, cristae architecture is central to mitochondrial energy conversion, as it organizes respiratory chain complexes into functional assemblies and supports localized proton gradients necessary for efficient electron transport. Disruption of this spatial organization is predicted to reduce respiratory efficiency even when respiratory complex abundance is preserved. In this context, reduced TACO1-dependent translation of cytochrome c oxidase subunit I likely constrains effective complex IV assembly or integration, limiting electron flux through the respiratory chain. Functional analyses support this interpretation, as TACO1 loss resulted in reduced basal and ATP-linked respiration, as well as diminished spare respiratory capacity, while maximal respiratory capacity remained relatively preserved. This pattern indicates inefficient electron transport rather than complete loss of respiratory machinery, a limitation that is likely to be particularly consequential in cardiomyocytes with high and fluctuating energetic demands.

Consistent with impaired mitochondrial performance, myocardial tissue exposed to chronic stress exhibited pronounced metabolic remodeling. Multivariate analyses revealed dominant shifts in metabolic profiles that exceeded inter-individual variability, suggesting convergence toward a shared adaptive state. Enrichment of amino acid, nucleotide, and pentose phosphate pathway-related metabolites indicates increased reliance on alternative substrates and redox buffering mechanisms when oxidative phosphorylation capacity is limited. Engagement of the pentose phosphate pathway is consistent with an increased requirement for NADPH-dependent antioxidant capacity, reflecting heightened oxidative stress. These coordinated metabolic changes support the interpretation that cardiac dysfunction arises from a regulated metabolic adaptation rather than an incidental collapse of energy metabolism. Structural remodeling of mitochondria paralleled these metabolic shifts, suggesting that architectural disruption of the organelle may precede, accompany, or reinforce metabolic reprogramming. However, analyses of human myocardial tissue alone could not definitively distinguish whether mitochondrial remodeling is driven primarily by chronic hemodynamic load, neurohormonal activation, or secondary disease processes. To address this limitation, we employed the mRen rat model, a genetically defined model of sustained renin angiotensin aldosterone system activation in which angiotensin II-driven hypertension serves as a dominant pathological stressor.

Within this model, we examined the contribution of angiotensin receptor signaling to augmented ganglionic synaptic plasticity. Angiotensin II exerts its effects through AT1 and AT2 receptors, which mediate excitatory and counterregulatory signaling, respectively, at sympathetic ganglia. Although increased excitatory signaling through AT1 receptors would be expected to enhance ganglionic long-term potentiation and sympathetic output, AT1 receptor density in superior cervical ganglia from hypertensive animals was comparable to that of normotensive controls. In contrast, AT2 receptor expression was significantly reduced at both transcript and protein levels. These findings indicate that loss of inhibitory angiotensin signaling, rather than increased excitatory receptor abundance, lowers the threshold for ganglionic plasticity and sympathetic activation in this model. Proteomic analyses of left ventricular tissue further supported a central role for renin angiotensin signaling in driving mitochondrial remodeling. Chronic activation of this pathway was associated with altered abundance of proteins involved in mitochondrial metabolism, redox balance, calcium handling, and cellular stress responses, including TACO1. Pharmacologic blockade with candesartan partially or completely normalized several of these changes, placing angiotensin II signaling upstream of the observed mitochondrial and proteostatic alterations. Notably, the regulation of TACO1 in this context and its relationship to complex IV function in human myocardium has not been previously reported.

Beyond effects on cristae structure and respiration, TACO1 loss resulted in redistribution of mitochondria toward distal cellular regions, a phenotype not observed with disruption of canonical fusion or cristae regulators. This finding suggests that TACO1 contributes to mitochondrial network positioning in addition to regulating inner membrane organization. Altered organelle positioning may influence local ATP delivery, calcium handling, and inter organelle communication, providing an additional mechanism through which mitochondrial remodeling can impair cellular function. At the molecular level, suppression of TACO1 was associated with activation of stress responsive pathways, including ATF4 and IRE1, consistent with engagement of integrated stress and proteostatic responses. Metabolomic and lipidomic analyses further indicated that broad changes in redox balance, amino acid metabolism, and membrane associated lipid species accompanied structural disruption of mitochondria. Alterations in lipid composition that support membrane curvature and stability may further influence cristae organization, reinforcing the link between mitochondrial structure and metabolic function.

In hiPSC-derived cardiomyocytes, TACO1 silencing resulted in mito-structural change and lipid accumulation. These imaging readouts are supportive of a model in which mitochondrial and metabolic remodeling contributes to repolarization changes. In the event of mitochondrial dysfunction there is a selective removal of damaged mitochondria through mitophagy. In siTACO1 hiPSC-CMs the lower mitochondrial count could reflect increased mitochondrial turnover, possibly through mitophagy. However, the current data do not directly assess mitophagic flux, so this is speculative. These observations support a framework in which chronic neurohormonal activation through RAAS modulation of TACO1, constraining mitochondrial translation of COX1, limiting effective complex IV function, and destabilizing cristae architecture.

Our observations across various models suggest a framework in which chronic neurohormonal activation through the renin angiotensin system modulates TACO1, limiting mitochondrial translation of cytochrome c oxidase subunit I, constraining complex IV function, and destabilizing cristae architecture. This structural and functional remodeling reduces respiratory efficiency and promotes oxidative stress, contributing to bioenergetic insufficiency and cardiac dysfunction. While the present data strongly supports this framework, direct causal relationships between individual steps remain to be fully defined.

Several limitations remain: it is not clear whether TACO1 directly influences cristae architecture or whether its effects are mediated entirely through altered respiratory complex assembly. The molecular mechanisms underlying TACO1 dependent regulation of mitochondrial positioning also require further investigation. In addition, although the integration of cellular, metabolic, and physiological data provides a multiscale view of disease progression, targeted *in vivo* manipulation will be required to establish the precise contribution of this pathway to cardiac function. In summary, this study reframes hypertensive heart failure as a disorder of translation dependent mitochondrial organization and identifies TACO1 as a regulatory node linking neurohormonal signaling to mitochondrial structure and bioenergetic performance. By extending current models of mitochondrial regulation beyond membrane dynamics to include translational control, these findings highlight mitochondrial protein synthesis as a critical axis through which systemic stress shapes cardiac energetics and disease progression.

## 4 Materials and Methods

### 4.1 All of Us Research Program Cohort Selection and Gene-Based PheWAS and LabWAS

#### 4.1.1 Study Cohort

Participants were selected from the *All of Us* Research Program, a U.S. longitudinal cohort established by the National Institutes of Health to advance precision medicine through enrollment of a large and diverse adult population aged ≥18 years. Enrollment was initiated in 2017, and all participants provided written informed consent, authorized access to their electronic health records (EHR), and completed baseline surveys capturing demographic information. Data were accessed through the *All of Us* Researcher Workbench (Controlled Tier) [24]. This study used Curated Data Repository version 8 (CDRv8; C2024Q3R4, released February 20, 2026), which includes participants enrolled and consented through October 1, 2023. Among 633,248 participants aged ≥18 years at consent, we identified individuals with available EHR data and short-read whole-genome sequencing (srWGS) data. Participants without EHR or srWGS data, or with assigned sex at birth other than male or female or missing/declined responses, were excluded. Rare predicted TACO1 loss-of-function (LoF) carriers were defined using variants annotated as frameshift, splice-acceptor, splice-donor, or stop-gained with allele frequency ≤0.01. Noncarriers met the same age-at-consent, EHR, srWGS, and sex-at-birth criteria and were excluded if they carried qualifying LoF variants in TACO1. The initial eligible case-control cohort comprised 314,519 participants; after derivation of regression covariates and exclusion of participants with missing covariate data, the final covariate-complete analytic sample included 180,076 participants, including 9 TACO1 LoF carriers and 180,067 noncarriers (Figure S1).

#### 4.1.2 Phenome-Wide Association Study (PheWAS)

We performed a gene-based PheWAS in the *All of Us* cohort to evaluate associations between TACO1 LoF carrier status and clinical phenotypes. Carrier status was encoded as the independent variable of interest. Covariates were derived using PheTK (v0.1.47), an *All of Us*-adapted Python framework for phecode-based phenome-wide association testing[25], and included age at last EHR event, sex at birth, and the first five genetic principal components. International Classification of Diseases, Ninth or Tenth Revision, Clinical Modification (ICD-9-CM/ICD-10-CM) diagnosis data were mapped to phecodes using phecode version 1.2 (U.S. ICD mapping). Logistic regression was performed for each phecode, with TACO1 LoF carrier status as the independent variable and phecode case status as the dependent variable. Analyses were restricted to phecodes with at least two occurrences on distinct dates and at least five cases. Statistical significance was assessed using Bonferroni correction based on the total number of phecodes tested[26]. In the TACO1 analysis, 1,607 phecodes were evaluated.

#### 4.1.3 Clinical Lab-Wide Association Scan (LabWAS)

We performed a clinical laboratory-wide association study (LabWAS)[27] in the *All of Us* cohort to identify laboratory phenotypes associated with TACO1 loss-of-function (LoF) carrier status. Laboratory measurements were extracted from the *All of Us* measurement table and harmonized across related concepts by mapping to shared laboratory phenotype labels, assigning each phenotype to a laboratory domain, and retaining the dominant unit for each phenotype. Repeated measurements were summarized at the participant level using the median value for each laboratory phenotype. Extreme values were excluded using a laboratory-specific outlier filter applied to participant-level median laboratory values (>4 standard deviations from the mean). Laboratory phenotypes were eligible for testing if available in at least 20 participants overall and at least three TACO1 LoF carriers. Laboratory values were rank-based inverse normal transformed and analyzed using linear regression, with TACO1 LoF carrier status as the independent variable of interest, adjusting for age at last EHR event, sex at birth, and the first five genetic principal components. In total, 45 laboratory phenotypes were tested. Nominal significance was defined as *p* < 0.05, and Bonferroni significance was defined as *p* < 0.05/45, corresponding to *p* < 1.11 × 10^−3^. The same covariate-complete analytic cohort (*n* = 180,076) was used for both PheWAS and LabWAS analyses (Figure S1).

### 4.2 Blood Pressure Measurements

Male mRen transgenic hypertensive rats (most commonly known as (mRen2)27) and Hannover Sprague Dawley (SD) rats, both 12–16 weeks, were acquired from Wake Forest University, Winston-Salem, NC. The animals were housed at East Carolina University, and all experiments were carried out in accordance with the guiding principles for the care and use of animals as mandated by the Institutional Animal Care and Use Committee. Candesartan cilexetil (purchased from Sigma-Aldrich (St. Louis, MO, USA)) was prepared by dissolving it in molecular biology grade DMSO to create a stock solution. This was further diluted in phosphate-buffered saline (PBS) to reach the target concentration, maintaining a final DMSO concentration of 1% (v/v). The solution was filter-sterilized using a 0.22 μm PVDF membrane filter before use. Rats (mRen+R_x_) were administered a daily dose of 16 mg/kg body weight via intraperitoneal injection for 28 consecutive days[28].

Blood pressures were recorded using the non-invasive blood pressure tail cuff method. Tail-cuff plethysmography is the least stressful on the animal and is low risk. This procedure allows rats to remain awake and takes only about 30 minutes per day. The rats are accustomed to the restraints during the first week and the process before the data collection periods. Rats were placed in acrylic restraints, and the occlusion and sensor cuffs were placed on the tail. In a dark, quiet, warm environment the tails were warmed, and 10 recordings were collected per rat per day for 28 days. Total systolic, diastolic, mean arterial pressure, heart rate, rate-pressure product, pulse pressure, and cardiac output recordings were averaged for SD and mRen animals. Tail-cuff BP measurements were performed in accordance with American Heart Association recommendations for BP assessment in experimental animals, and reliability was supported by repeated daily measurements following acclimation procedures[29].

### 4.3 Non targeted Proteomics analysis

#### 4.3.1 Peptide Isolation

Left ventricular tissue samples were manually minced using scalpels and resuspended in 500 μL lysis buffer (50 mM Tris, pH 8.0, 0.5% sodium deoxycholate, 40 mM NaCl, 2 mM MgCl_2_). Samples were sonicated on ice with a microtip (10 s bursts at 30% amplitude), with at least three sonication cycles performed. The samples were then subjected to a freeze–thaw cycle at −80 °C overnight. Proteins were isolated by ice-cold methanol precipitation (2:1, v/v; methanol: sample) at −20 °C overnight. Protein pellets obtained by centrifugation were washed twice with ice-cold methanol (300 μL each wash). Isolated proteins were purified using the PreOmics iST kit and fractionated with the PreOmics fractionation add-on kit according to the manufacturer’s instructions, yielding three fractions per sample. In total, 45 LC–MS/MS runs were acquired, with each fraction analyzed as an individual run but later combined and interpreted as a single biological sample. For peptide purification, fractions were dried under a nitrogen (N_2_) stream. Dried peptides were reconstituted to a concentration of 0.25 mg/mL in loading buffer (98:2 water:acetonitrile with 0.1% formic acid). Protein concentration was determined using a NanoDrop spectrophotometer, and 1 μg total peptide (4 μL) from each fraction was injected for LC–MS/MS analysis.

#### 4.3.2 LC-MS/MS for Proteomics

Peptides were analyzed by nanoLC-MS/MS using an UltiMate 3000 RSLCnano system (ThermoFisher) coupled to a Q Exactive Plus Hybrid Orbitrap mass Spectrometer (ThermoFisher) via nanoeslectrospray ionization. Peptides were separated using an effective linear gradient of 4–35% acetonitrile (0.1% formic acid) over 120 min. For data dependent acquisition, MS spectra were acquired in positive mode with an NCE of 32. MS1 were performed at a resolution of 70,000 with an AGC target of 1×10^5^ ions and a maximum injection time of 100 ms. MS2 spectra were collected on the top 20 most abundant precursor ions with a charge >1 using an isolation window of 1.5 m/z and fixed first mass of 140 m/z. The normalized collision energy for MS2 scans was 30. MS2 spectra were acquired at 17,500 resolutions with a maximum injection time of 60 ms, an AGC target of 5×10^4^ and a dynamic exclusion of 20 sec.

#### 4.3.3 Database Searching and Analysis

FragPipe (v 22.0,) was used for raw data analysis with default search parameters for open and Label-Free Quantification Matching Between Runs (LFQ-MBR) workflows[30]. All samples were searched together with biological replicates (n=5) for group identified. An initial open search against the canonical Uniprot Rattus norvegicus reference proteome (UP000234681, accessed 9/2024) was used to identify potential post-translational modifications for inclusion in the LFQ-MBR workflow. Precursor m/z tolerance was set to −150 to 500 Da and fragment tolerance was ±20 ppm with 3 missed cleavages for Tryp and Lys-C allowed. Peptide spectrum matches (PSMs) were validated using PeptideProphet and results were filtered at the ion, peptide, and protein level with a 1% false discovery rate (FDR). Based on these initial searches, the following variable modifications were included in the LFQ-MBR analysis: oxidation (+15.5995 Da on Met), deamidation (+0.98401 Da on Gln and Asn), acetylation (+42.0106 Da on N-terminus), loss of ammonia (−18.0106 Da on Glu) and fixed modification carbamodiomethyl (+57.025 Da on Cys).

For LFQ-MBR analysis, data were search against the canonical Uniprot Rattus norvegicus reference proteome (UP000234681, accessed 9/2024). Precursor ion m/z tolerance was ±20 ppm with 3 missed cleavages for Trypsin/LysC allowed. The search results were filtered using a 1% FDR at the ion, peptide, and protein levels. PSMs were validated using Percolator and label free quantification was carried out using IonQuant[31]. Match between runs FDR rate at the ion level was set to 10% for the top 300 runs. Proteins with >95% probability of ID, ≥1 unique peptides, and in more than 80% of a sample group (i.e. 4/5 injections) were considered high confidence IDs and retained for analysis. Intensities were log2-transformed, quantile-normalized within the entire sample run, and relative abundances for low-sampling proteins were determined via k-NN methods (15 neighbors) in Perseus[32].

### 4.4 Immunofluorescence Staining

Frozen rat SCGs were sectioned into 5-μm-thick sections and mounted onto glass slides. Slides were allowed to air-dry at room temperature and were then fixed in 4% paraformaldehyde in 0.1 M phosphate (PO_4_) buffer for 5 minutes. Tissues were permeabilized with 0.2% Triton X-100 and washed three times with 1× PBS for 10 minutes each.

Each specific target antibody was added and incubated overnight in a humidifying chamber in the dark at 4°C. The fluorescent targets of their antibody dilutions are as follows: anti-angiotensin II receptor type 1 (AT_1_) extracellular-ATTO Fluor-550 Antibody (AAR-011-AO Alomone Labs, 1:50 dilution), anti-angiotensin II receptor type 2 (AT_2_) extracellular-ATTO Fluor-488 Antibody (AAR-012-AG Alomone Labs, 1:50 dilution), and in-house labeled Fluor-488 tagged MAS (1:50 dilution).

After three additional washes with 1×Phosphate Buffer Solution, tissue sections were mounted in ProLong Diamond antifade mounting medium containing the nuclear stain 4′,6-diamidino-2-phenylindole (Thermo Fisher Scientific) and examined using a confocal microscope (Zeiss LSM 800). Photon exposure settings were kept constant for image acquisition from both SD and mRen specimens. The cytoskeletal marker Alexa Fluor 594 Phalloidin was used in both groups, and primary antibodies were detected via their conjugated fluorescent probes.

For quantitative imaging, four to six biological replicates per group [i.e., SD vs. mRen; n = 4–6] were analyzed for each target, and three to four fields per SCG section were acquired. SCG tissue thickness ranged from 0.1 to 0.2 mm, and care was taken to avoid overlap between images during acquisition[33].

### 4.5 Serial Block-Face Scanning Electron Microscopy and Three-Dimensional Reconstruction

For three-dimensional ultrastructural analysis, cardiac tissue was processed for SBF-SEM following a heavy-metal contrast enhancement protocol adapted from established methods[34]. Briefly, tissue was fixed in 2% glutaraldehyde in 0.1 M cacodylate buffer, immersed sequentially in 3% potassium ferrocyanide with 2% osmium tetroxide (1 hour, 4°C), 0.1% thiocarbohydrazide (20 minutes), 2% osmium tetroxide (30 minutes), and 1% uranyl acetate overnight at 4°C, with deionized water washes between each step. Samples were then immersed in 0.6% lead aspartate (30 minutes, 60°C), dehydrated through graded acetone, infiltrated with TAAB 812 hard resin, and polymerized at 60°C for 36–48 hours. Blocks were trimmed to 0.5 mm × 0.5 mm and mounted on aluminum pins for acquisition on an FEI/Thermo Scientific Volume Scope 2 SEM. Between 300 and 400 serial sections at a slice thickness of 0.09 μm were acquired per block. Manual contour segmentation of the outer mitochondrial membrane was performed across consecutive orthoslices, and three-dimensional surface meshes were rendered using Amira software (Thermo Fisher Scientific), following validated protocols [34–35].

The following 3D morphometric parameters were extracted from closed triangulated surface meshes generated in Amira:

#### 4.5.1 Mitochondrial Volume (V):

Mitochondrial volume was calculated from the closed 3D surface mesh as the total space enclosed by the triangulated surface:

$$V=\sum_{i=1}^{N}v_{i}$$

where v_i represents the volume of each tetrahedral element composing the enclosed surface mesh and N is the total number of elements. Equivalently, when derived from voxel-based segmentation:

$$V=N_{vox}\times (x\cdot y\cdot z)$$

where N_vox is the total number of voxels assigned to a given mitochondrion and x, y, z corresponds to calibrated voxel dimensions in each spatial axis. All volumes are reported in cubic micrometers (μm^3^).

#### 4.5.2 Mitochondrial Surface Area (A):

Surface area was extracted as the sum of the areas of all triangular faces composing the 3D mesh:

$$A=\sum_{i=1}^{M}A_{i}$$

where A_i represents the area of each triangular face and M is the total number of faces. Values are reported in square micrometers (μm^2^).

#### 4.5.3 Mitochondrial Perimeter:

Perimeter was quantified from 2D EM cross-sections by manually tracing the outer mitochondrial membrane and computing the total contour length:

$$P=\sum_{j=1}^{K}l_{j}$$

where l_j represents each contiguous segment of the mitochondrial profile and K is the total number of segments. Values are averaged across sections and reported in micrometers (μm).

#### 4.5.4 Sphericity (Φ):

Mitochondrial shape was assessed independently of size using sphericity, calculated from volume and surface area as:

$$\Phi =\frac{\pi^{1/3}(6V{)}^{2/3}}{A}$$

A sphericity value of 1.0 indicates a perfect sphere, with lower values indicating greater elongation or structural complexity[34a, 36].

#### 4.5.5 Mitochondrial Complexity Index (MCI):

Mitochondrial branching and morphological complexity were quantified using the three-dimensional MCI, a surface-to-volume-based metric that captures deviations from simple geometric shapes arising from fusion events, network formation, and nanotunnel development, and is invariant to absolute mitochondrial size[36–37].

MCI was initially defined as:

(1) $$MCI=\frac{SA^{3/2}}{4\pi V}$$

where SA is mitochondrial surface area (μm^2^) and V is mitochondrial volume (μm^3^). The fractional exponent (3/2) renders the expression dimensionless. To expand dynamic range and improve sensitivity to differences in highly branched mitochondria, the expression was squared, yielding the final formulation:

(2) $$MCI={\left(\frac{SA^{3/2}}{4\pi V}\right)}^{2}=\frac{SA^{3}}{16\pi^{2}V^{2}}$$

Equations (1) and (2) encode identical structural information; Equation (2) expands the dynamic range and provides a more intuitive correspondence to observed morphological differences[36]. Low MCI values indicate compact, near-spherical mitochondria with minimal branching; high MCI values indicate elongated, branched, or highly networked structures. MCI is unbounded, allowing complexity to scale without an upper limit as mitochondrial architecture becomes increasingly elaborate.

### 4.6 Transcriptomic and Lipid Analyses

#### 4.6.1 Human myocardial Tissue samples

Heart Tissue samples from the left ventricle were obtained from donors who served as controls and subjects diagnosed with non-ischemic cardiomyopathy or ischemic cardiomyopathy. Deidentified samples were processed in accordance with the IRB protocol and ethical guidelines. Personal identifier information, including age, sex, and diagnosis, is summarized in Figure 3(A). Samples were quickly flash frozen at the time of collection and stored at −80 °C until processing.

#### 4.6.2 For Lipid and Metabolite Extraction

Frozen myocardial tissue was weighed and homogenized under liquid nitrogen using a cryogenic mill. Metabolites were extracted using cold organic solvent extraction optimized for polar and semi-polar metabolites, as adapted from previously described protocols. Briefly, tissue was extracted in pre-chilled solvent (methanol: acetonitrile: water–based extraction), vortexed, incubated on dry ice to quench metabolic activity, and centrifuged at high speed to remove insoluble debris. Supernatants were collected and stored at −80 °C before analysis. Internal standards were included to control for extraction efficiency, derivatization variability, and instrument drift[38].

#### 4.6.3 GC-MS–based transcriptome

Extracted lipids were derivatized and analyzed by gas chromatography–mass spectrometry (GC-MS) at a dedicated high-resolution mass spectrometry facility using standardized operating procedures. Metabolites were identified by comparison of retention time and mass spectral signatures against an in-house and commercially validated reference library. Raw spectra were processed using vendor-compatible software and exported for downstream analysis. Peak areas were normalized to internal standards and tissue input to account for extraction and loading differences.

#### 4.6.4 Multivariate and univariate transcriptomic analysis

Processed metabolomic datasets were analyzed using MetaboAnalyst (v5.0), following approaches consistent with metabolomics standards. Data were log-transformed and auto-scaled before analysis. Principal component analysis (PCA) was used for unsupervised dimensionality reduction and visualization of global metabolic differences. One-way ANOVA was applied to identify significantly altered metabolites across diagnostic groups. Metabolites with P < 0.05 were considered significantly altered and retained for pathway analysis.

### 4.7 Human induced pluripotent stem cell-derived ventricular cardiomyocytes culturing

Human induced pluripotent stem cell-derived ventricular cardiomyocytes (hiPSCs) were derived from dermal fibroblasts and donated by the laboratory of Dr Joseph Wu (Stanford University, CA, USA). Id1-overexpressing hiPSCs were dissociated with 0.5 mM EDTA (Thermo Fisher Scientific) in PBS without CaCl_2_ and MgCl_2_ (Corning) at for 37°C 7 min. hiPSCs were resuspended in mTeSR-Plus medium (StemCell Technologies) supplemented with 2 μM thiazovivin (StemCell Technologies) and plated in a Matrigel-coated 12-well plate at a density of 3×10^5^ cells per well and cultured for 2 days, until they reached ≥90% confluence to begin ventricular cardiomyoctye differentiation. At day 0, cells were treated with 6 μM CHIR99021 (Selleck Chemicals) in S12 medium. After 48h (day 2), cells were treated with 2 μM Wnt-C59 (Selleck Chemicals) in S12 medium. After 48h (day 4) media was replaced with S12 medium for 24h. At day 5, cells were dissociated with TrypLE Express (Gibco) for 2 min and blocked with RPMI (Gibco) supplemented with 10% fetal bovine serum (FBS; Omega Scientific). Cells were resuspended in S12 medium supplemented with 4 mg/l Recombinant Human Insulin (Gibco) (S12+ medium), and 2 μM thiazovivin and plated onto a Matrigel-coated 12-well plate at a density of 9×10^5^ cells per well. The S12+ medium was changed at day 8 and replaced at day 10 with Albumax media containing RPMI (Gibco) supplemented with 213 μg/μl L-ascorbic acid (Sigma-Aldrich), 500 mg/l BSA-FV (Gibco), 0.5 mM L-carnitine (Sigma-Aldrich) and 8 g/l AlbuMAX Lipid-Rich BSA (Gibco). At day 15, cells were purified with lactate medium [RPMI without glucose, 213 μg/μl L-ascorbic acid, 500 mg/L BSA-FV and 8 mM sodium-DL-lactate (Sigma-Aldrich)], for 4 days. At day 19, the medium was replaced with Albumax medium.

### 4.8 siRNA Transfection

Cells (HEK and HeLa) were transfected with small interfering RNAs (siRNAs) targeting TACO1, OPA1, or MFN2, or with a non-targeting control siRNA, using a commercial lipid-based transfection reagent (e.g., Lipofectamine RNAiMAX, Thermo Fisher Scientific) according to the manufacturer’s instructions. Briefly, siRNA was diluted to a final concentration of 10–25 nM in Opti-MEM reduced-serum medium and incubated with the transfection reagent for 20 minutes at room temperature before addition to cells. Cells were incubated for 48–72 hours post-transfection before downstream analysis. Knockdown efficiency was confirmed by quantitative RT-PCR.

For hiPSC-derived VCMs at day 25 of differentiation were dissociated with TrypLE Select 10X (Thermo Fisher Scientific) for 8 min, and TrypLE Select 10X was neutralized by adding RPMI supplemented with 10% FBS. Cells were resuspended in Albumax media supplemented with 2 μM thiazovivin and plated at a density of 6000 cells per well in a Matrigel-coated 384-well plate and cultured for 2 days to day 27 of differentiation. On day 27, SiRNA knockdown of TACO1 was performed by removing all Albumax media and replacing it with 100μl of OPT-MEM (Gibco) supplemented with 1μl RNAiMAX (Invitrogen) and 5μl siRNA at 100μM for 4 hours, before being replaced with Albumax media. Cells were cultured for a further 24h before functional calcium handling assays[39].

### 4.9 Calcium assay in hiPSCs

Calcium handling assay was performed using the labeling protocol described in Kervadec (2023). Briefly, cells were first washed with pre-warmed Tyrode’s solution (Sigma-Aldrich) by removing 50 μl medium and adding 50 μl Tyrode’s solution five times using a multichannel pipette. After the fifth wash, 50 μl of 2× dye solution consisting of calcium-sensitive dye Cal520 AM (ab171868, 1:10,000) diluted in Tyrode’s solution supplemented with 0.2 μl of 10% Pluronic F127 (diluted in water; Thermo Fisher Scientific), 1mL assay buffer (Invitrogen), 100μl probenecid F-127 (Invitrogen) and 20 μg/ml Hoescht 33258 (diluted in water; Thermo Fisher Scientific) was added to each well. Wells that had been treated with drugs were given the CAL520 cocktain which was supplemented with the appropriate well. The plate was placed back in the 37°C 5% CO_2_incubator for 1h. After the incubation time, cells were washed four times with fresh pre-warmed Tyrode’s solution. hiPSC-derived ACMs were then automatically imaged with an Image Xpress Micro XLS microscope at an acquisition frequency of 100 Hz for a duration of 10s with an excitation wavelength of 485/20 nm and emission filter 525/30 nm. A single image of Hoescht 33258 was acquired before the time series. Fluorescence over time quantification and trace analysis were performed using custom software packages developed by Molecular Devices and the Colas laboratory[39].

### 4.10 Morphological Profiling

hiPSCs were culture to day 25 as previously described and replated in 384-well plates at a density of 6000 cells per well. Cells were treated with SiRNAs and drugs as previously described. Mitotracker Orange CM-H2TMRos (150nM) was added to the cells 30 minutes before the end of the drug treatment. Prior to fixation, cells were washed four times with pre-wared Tyrode solution and were fixed with 4% PFA for 10 minutes. Cells were then permeabilized with 0.1% Triton X in PBS for 10 minutes and washed four times with PBS. Cells were then treated with blocking buffer was added for 1 hour. Cells were stained with α-Actinin (mouse; A7811, Invitrogen) primary antibody in blocking buffer overnight at 4°C, then were washed 6 times with PBS at room temperature before being stained with secondary antibody conjugated with a 488 fluorophore (Donkey anti-mouse; A21202, Invitrogen) and DAPI for 1h at room temperature, washed four times with PBS. LipidTOX (1:1000 dilution) was added to the cells for 30 minutes at room temperature and then were imaged using an Image Xpress Micro XLS microscope at 20X. Images were analyzed using INCarta software (Molecular devises) using a pretrained SINAP module to accurately segment mitochondria and a robust puncta algorithm was used for the detection of lipid droplets. Statistical analysis was performed on the extracted data using a one-Way ANOVA followed by a Dunn’s post-hoc test[39].

### 4.11 Transmission Electron Microscopy

For ultrastructural analysis of mitochondria, cells were fixed in 2.5% glutaraldehyde in 0.1 M sodium cacodylate buffer (pH 7.4) for 1 hour at room temperature, followed by post-fixation in 1% osmium tetroxide for 1 hour on ice. Samples were then dehydrated through a graded ethanol series and embedded in epoxy resin. Ultrathin sections (70–90 nm) were cut using an ultramicrotome, mounted on copper grids, and stained with uranyl acetate and lead citrate. Sections were imaged using a transmission electron microscope at an appropriate accelerating voltage. All imaging was performed blinded to experimental condition. Mitochondrial length and cross-sectional area were measured from TEM images using ImageJ/FIJI software. Individual mitochondria were manually traced, and measurements were recorded for a minimum of 50–100 mitochondria per condition across multiple cells and biological replicates. Data are presented as mean ± SEM.

### 4.12 Quantification of Cristae Ultrastructure

Cristae morphology was assessed from TEM images and scored across three parameters: cristae score, cristae area, and cristae volume. Cristae score was assigned on a semi-quantitative scale reflecting overall cristae complexity and density. Cristae area and volume were measured by manual segmentation of individual cristae structures within mitochondrial cross-sections using ImageJ/FIJI. A minimum of 30–50 mitochondria per condition were analyzed across multiple independent experiments. A blinded observer performed all quantification. Data are presented as mean ± SEM.

### 4.13 Mitochondrial Membrane Potential Assay

Mitochondrial membrane potential was assessed using a fluorescent indicator sensitive to membrane potential (e.g., JC-1 or TMRM), following the manufacturer’s protocol. Where indicated, cells were treated with 200 μM hydrogen peroxide (H_2_O_2_) as a positive control for membrane potential dissipation, and/or with 4-BPA (concentration as determined empirically) as a mitochondrial protective agent. Following dye loading, cells were washed and imaged by fluorescence microscopy. Fluorescence intensity was normalized to cell area and expressed relative to the control condition. A minimum of 10 cells per condition were quantified per experiment across at least three independent biological replicates. Data are presented as one-way ANOVA made mean ± SEM, and statistical comparisons with post-hoc multiple comparisons testing.

### 4.14 Live-Cell Imaging and 3D Mitochondrial Morphology

HEK and HeLa cells were cultured at a density of 50,000 cells per dish and allowed to adhere overnight. Following visual confirmation of adherence, cells were subjected to the indicated treatment conditions. For HEK cells, mitochondrial morphology was assessed following treatment with the MFN2 inhibitor Mfi8 (MedChem Express, Catalog No. HY-150031) at 20 μM for 6 hours, the OPA1 inhibitor MYLS22 (Millipore Sigma, Catalog No. AMBH97B9F609) at 50 μM for 48 hours, the MICOS inhibitor Miclxin (MedChem Express, Catalog No. HY-138301) at 30 μM for 1 hour, or DMSO vehicle control. For HeLa cells, mitochondrial morphology was assessed following siRNA-mediated knockdown of TACO1, MiCOS, OPA1, or MFN2, or transfection with a non-targeting control siRNA, as described above.

Live-cell mitochondrial dynamics were visualized using a Nikon Eclipse Ti2 inverted fluorescence microscope equipped with a Yokogawa CSU-W1 spinning disk confocal scanner, Hamamatsu Fusion BT camera, SoRa super-resolution module, environmental chamber, piezo stage controller, and solid-state lasers (405, 488, 561, and 640 nm), all controlled via NIS-Elements AR software (version 5.42). Cells cultured in 35-mm MatTek glass-bottom dishes were labeled with MitoTracker Orange and imaged using a 100× Plan Apo Lambda D oil immersion objective (NA 1.45) through the 561 nm channel at 2.5-second intervals over 5 minutes in either standard W1 mode (xy pixel size: 65 nm) or SoRa super-resolution mode (xy pixel size: 23 nm). Laser power was maintained at 5% with a 100 ms exposure time per frame to minimize phototoxicity while preserving signal quality (SNR ~1.5–2.5). Z-stability was maintained throughout all acquisitions using the Perfect Focus System. High-resolution z-stacks (10–20 μm thick) were acquired in SoRa mode with 100 nm step sizes and subsequently processed using the Nikon Batch Deconvolution module (version 6.10.02), implementing Blind and Richardson-Lucy deconvolution algorithms with 20 iterations and automatic noise estimation. SoRa imaging and image processing were performed, in part, through the Vanderbilt Cell Imaging Shared Resource and Nikon Center of Excellence.

### 4.15 Mitochondrial Volume, Surface Area, and Morphology

Mitochondria were segmented using the Imaris Surface module, which identifies individual mitochondria and interconnected mitochondrial networks as discrete surface objects. Image background subtraction was performed at 37.1 μm. Surface generation was performed using a Gaussian smoothing filter with a width of 0.129 μm to reduce high-frequency noise before segmentation. Segmentation was carried out using the Imaris machine-learning pixel-classification workflow to improve the separation of mitochondrial signal from background. The trained classifier was generated using representative images and applied uniformly across all samples without further adjustment. Objects smaller than 121 voxels were excluded to remove noise and sub-resolution structures. Mitochondrial area, volume, and sphericity were extracted from segmented surface objects and compared across conditions. Data are presented as violin plots representing individual mitochondrial measurements pooled across multiple cells and biological replicates. Statistical significance was assessed using one-way ANOVA with post-hoc multiple comparisons, with significance set at p < 0.0001.

### 4.16 Concentric Spatial Analysis of Mitochondrial Distribution

Oxygen consumption rate (OCR) was measured using a Seahorse XF Analyzer (Agilent Technologies) with the Mito Stress Test kit according to the manufacturer’s instructions. Briefly, control siRNA- or TACO1 siRNA-transfected cells were seeded in Seahorse XF cell culture microplates at an appropriate density 24 hours prior to the assay. On the day of the assay, cells were equilibrated in Seahorse XF DMEM assay medium supplemented with 10 mM glucose, 1 mM pyruvate, and 2 mM glutamine (pH 7.4) for 1 hour at 37°C in a non-CO_2_ incubator. Sequential injections of oligomycin (ATP synthase inhibitor), FCCP (uncoupler), and rotenone/antimycin A (complex I/III inhibitors) were used to determine basal OCR, ATP-linked OCR, maximal OCR, and spare respiratory capacity. OCR values were normalized to total protein content per well as determined by BCA assay. Data are presented as mean ± SEM from a minimum of three independent experiments. Statistical significance between control and TACO1 knockdown conditions was assessed by unpaired two-tailed Student’s t-test.

### 4.17 Mitochondrial Respiration (Seahorse XF Assay)

Oxygen consumption rate (OCR) was measured using a Seahorse XF Analyzer (Agilent Technologies) with the Mito Stress Test kit according to the manufacturer’s instructions. Briefly, control siRNA- or TACO1 siRNA-transfected cells were seeded in Seahorse XF cell culture microplates at an appropriate density 24 hours prior to the assay. On the day of the assay, cells were equilibrated in Seahorse XF DMEM assay medium supplemented with 10 mM glucose, 1 mM pyruvate, and 2 mM glutamine (pH 7.4) for 1 hour at 37°C in a non-CO_2_ incubator. Sequential injections of oligomycin (ATP synthase inhibitor), FCCP (uncoupler), and rotenone/antimycin A (complex I/III inhibitors) were used to determine basal OCR, ATP-linked OCR, maximal OCR, and spare respiratory capacity. OCR values were normalized to total protein content per well as determined by BCA assay. Data are presented as mean ± SEM from a minimum of three independent experiments. Statistical significance between control and TACO1 knockdown conditions was assessed by unpaired two-tailed Student’s t-test.

### 4.18 Statistical Analysis

Data are represented as mean ± standard error of the mean with individual data points represented. For two-group comparisons, unpaired two-tailed Student’s t-tests were used. For multiple-group comparisons, one-way analysis of variance followed by Fisher’s protected least significant difference test was used. For distributional analysis of APD75 population data, two-sample Kolmogorov-Smirnov tests were used. All statistical tests were done with GraphPad Prism. The symbols *p < 0.05, **p < 0.01, ***p < 0.001, and ****p < 0.0001 indicate statistical significance. A minimum of three biological replicates were used for all experiments.

## Supplementary Material

Supplement 1

Supplementary Files

This is a list of supplementary files associated with this preprint. Click to download.

• 2TACO1Supplement.docx

## Data Availability

The data that support the findings of this study are available from the corresponding author AJH upon reasonable request. To protect participant privacy, all *All of Us* data used in this study are available only to registered researchers through the *All of Us* Researcher Workbench, which can be accessed at https://workbench.researchallofus.org/login, subject to institutional agreements (e.g., a Data Use and Registration Agreement), required training, and compliance with the data use policy. Analysis code can be shared with authorized *All of Us* Researcher Workbench users upon reasonable request. All *All of Us* data used in this study are available only to registered researchers through the *All of Us* Researcher Workbench, which can be accessed at https://workbench.researchallofus.org/login, subject to institutional agreements (e.g., a Data Use and Registration Agreement), required training, and compliance with the data use policy. Proteomics datasets generated in this study will be deposited to the ProteomeXchange consortium via the jPOST partner repository and will be released publicly upon publication. Repository accession numbers and persistent links will be provided in the final published article.
