# Supplementary material for: TACO1 regulates mitochondrial adaptation in hypertension-induced cardiac remodeling and heart failure
Source: Res Sq. 2026 May 5:rs.3.rs-9589283. Preprint. [Version 1] doi: 10.21203/rs.3.rs-9589283/v1 (PMC13174799; doi:10.21203/rs.3.rs-9589283/v1)
Supplement: Supplement 1 [file NIHPPRS9589283V1-supplement-1.pdf]

Supporting Information

Supporting Information is available from the Wiley Online Library or from the author.

Hypertensive AT1R signaling suppresses mitochondrial translation control.

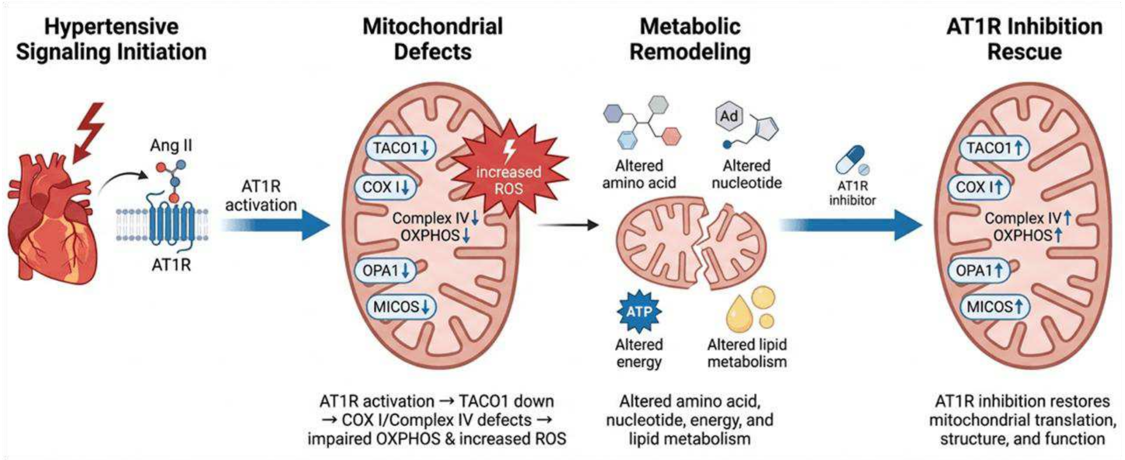

Angiotensin II mediated suppression of TACO1 mediated COX1 synthesis, limiting complex IV efficiency, destabilizing cristae architecture and elevating oxidative stress. AT1R blockade reverses COX1 translation, improves complex IV performance, and re-establishes inner-membrane organization.

Supporting Information

TACO1 regulates mitochondrial adaptation in hypertension-induced cardiac remodeling and heart failure.

Berwin Singh Swami Vetha[1,2†], Ronald McMillan[3†], James Marchant[4†], Mohd Mabood Khan[5], Joyonna Gamble-George[6,7,8,9], Jeremiah Afolabi[10], Edgar Garza-Lopez[11], Andrea G. Marshall[12], Calixto Pablo Hernandez Perez[6], Dillon Garbrandt[1], Bret Mobley[12], Jenny Schafer[13], Max Kushner[13], Oleg Kovtun[14], Debra D. Murray[15], Tonya Zeczycki[16], Annet Kirabo[10,17], Alexandre Colas[4], Celestine Wanjalla[2], Dao-Fu Dai[18], Melanie McReynolds[19], Azeez Aileru[1*], Antentor Hinton Jr.[6*]

[†]Co-first authors, [*]Co-corresponding authors and co-senior authors.

[1]Department of Foundational Science, East Carolina University, Greenville, NC, USA
[2]Department of Pharmacology and Toxicology, East Carolina University, Greenville, NC, USA
[3]Department of Medicine, Division of Infectious Diseases, Vanderbilt University Medical Center, Nashville, TN, USA
[4]Development, Aging and Regeneration Program, Sanford Burnham Prebys Medical Discovery Institute, La Jolla, CA, USA
[5]Department of Medicine, Division of Genetic Medicine & Clinical Pharmacology, Vanderbilt University Medical Center, Nashville, TN, USA
[6]Department of Computer Science, Whiting School of Engineering, Johns Hopkins University, Baltimore, MD 21218, USA
[7]Department of Epidemiology, Harvard T.H. Chan School of Public Health, Boston, MA 02115, USA
[8]Department of Social and Behavioral Sciences, Yale School of Public Health, New Haven, CT, 06520, USA
[9]Environmental Sciences Graduate Program, Oregon State University, Corvalis, OR 97331, USA
[10]Department of Molecular Physiology and Biophysics, Vanderbilt University, Nashville, TN, USA
[11]Department of Internal Medicine, University of Iowa, Iowa City, IA, USA
[12]Department of Pathology, Vanderbilt University Medical Center, Nashville, TN, USA
[13]Department of Cell and Developmental Biology, Vanderbilt University, Nashville, TN, USA
[14]Department of Chemistry, Vanderbilt University, Nashville, TN, USA
[15]Department of Molecular and Human Genetics, Baylor College of Medicine, Houston, TX, USA
[16]Department of Biochemistry and Molecular Biology, East Carolina University, Greenville, NC, USA
[17]Vanderbilt Institute for Global Health, Vanderbilt University Medical Center, Nashville, TN, USA
[18]Department of Pathology, Johns Hopkins University School of Medicine, Baltimore, MD, USA
[19]The Huck Institutes of the Life Sciences; Department of Biochemistry and Molecular Biology, Pennsylvania State University, University Park, PA, USA

Co-corresponding authors and co-senior authors.

Antentor Hinton Jr., PhD
Department of Molecular Physiology & Biophysics
Vanderbilt University, Nashville, TN, USA
Email: antentor.o.hinton.jr@vanderbilt.edu.

Azeez Aileru., PhD
Department of Foundational Science,
East Carolina University, Greenville, NC, USA
Email: ailerua19@ecu.edu

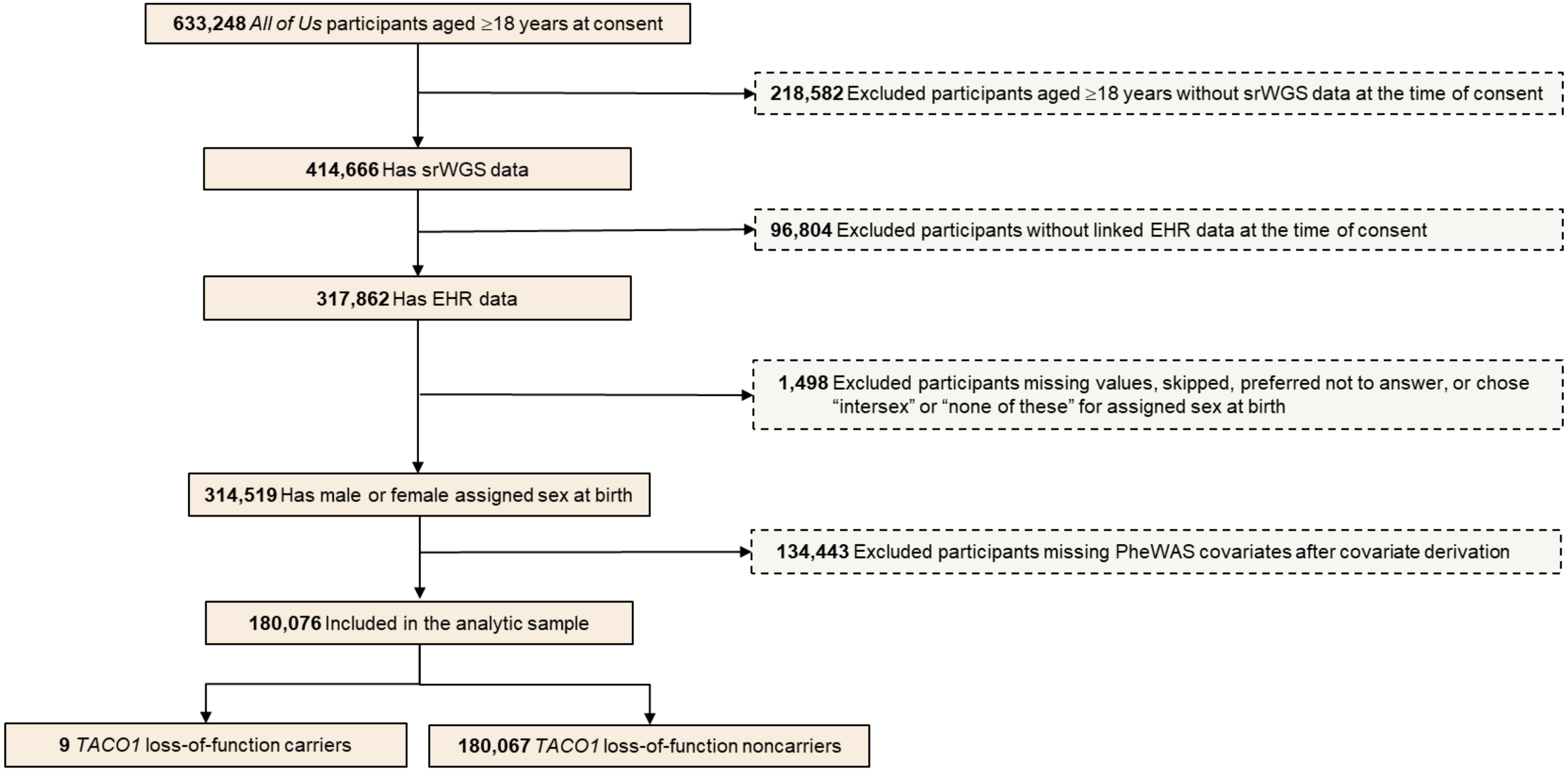

Figure S1. Flowchart of *TACO1* loss-of-function carrier cohort selection for covariate-adjusted PheWAS analysis from the *All of Us* Research Program. Participants were selected from the *All of Us* Research Program using Controlled Tier Dataset version 8, including participants enrolled and consented through October 1, 2023. Among 633,248 participants aged ≥18 years at consent, participants without srWGS data, without linked EHR data, or with assigned sex at birth other than male or female or missing and declined responses were excluded. *TACO1* rare predicted LoF carriers were defined using variants annotated as frameshift, splice-acceptor, splice-donor, or stop-gained, with allele frequency ≤0.01. This yielded an initial eligible case-control cohort of 314,519 participants, including 24 *TACO1* LoF carriers and 314,495 noncarriers. After derivation of regression covariates and removal of participants with missing covariate data, the final covariate-complete analytic sample comprised 180,076 participants, including 9 *TACO1* LoF carriers and 180,067 noncarriers (controls). srWGS, short-read whole-genome sequencing; EHR, electronic health record; LoF, loss-of-function.

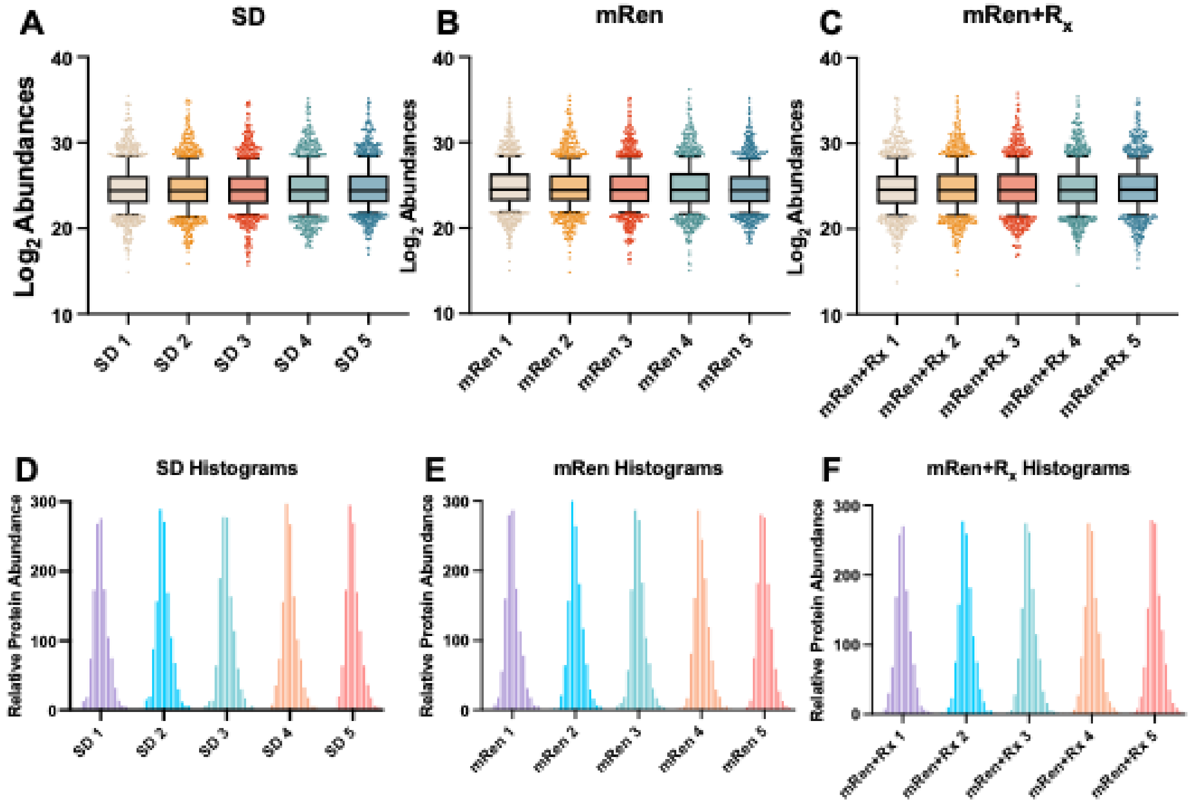

Figure S2: Protein log₂ abundance (A-C) & relative abundance in heart Left Ventricle tissue (D-F).

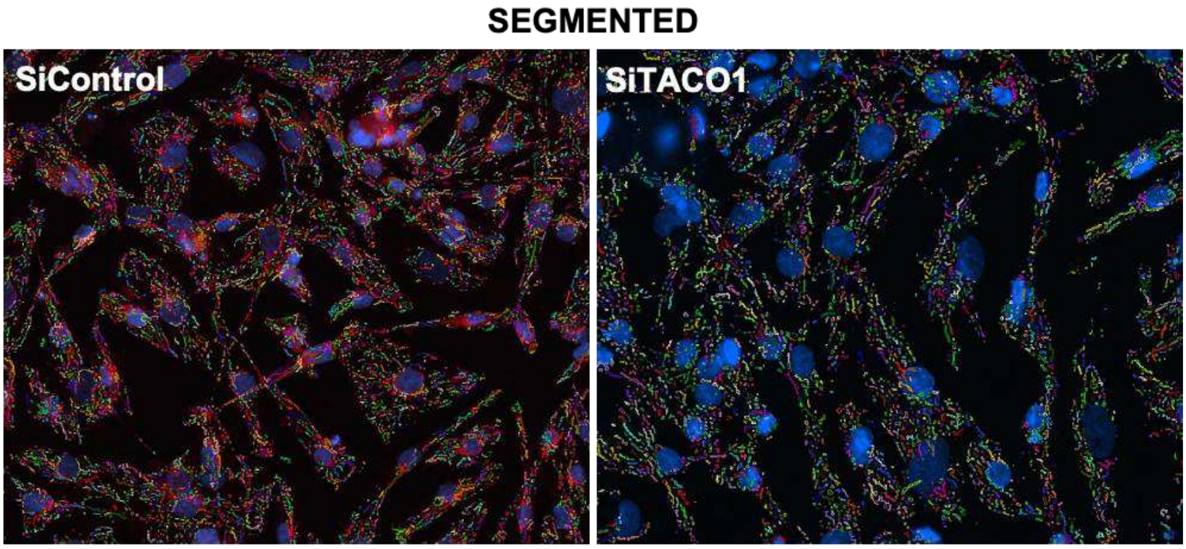

Figure S3: Ventricular-Like hiPSCs Mitochondrial segmentation imaging.

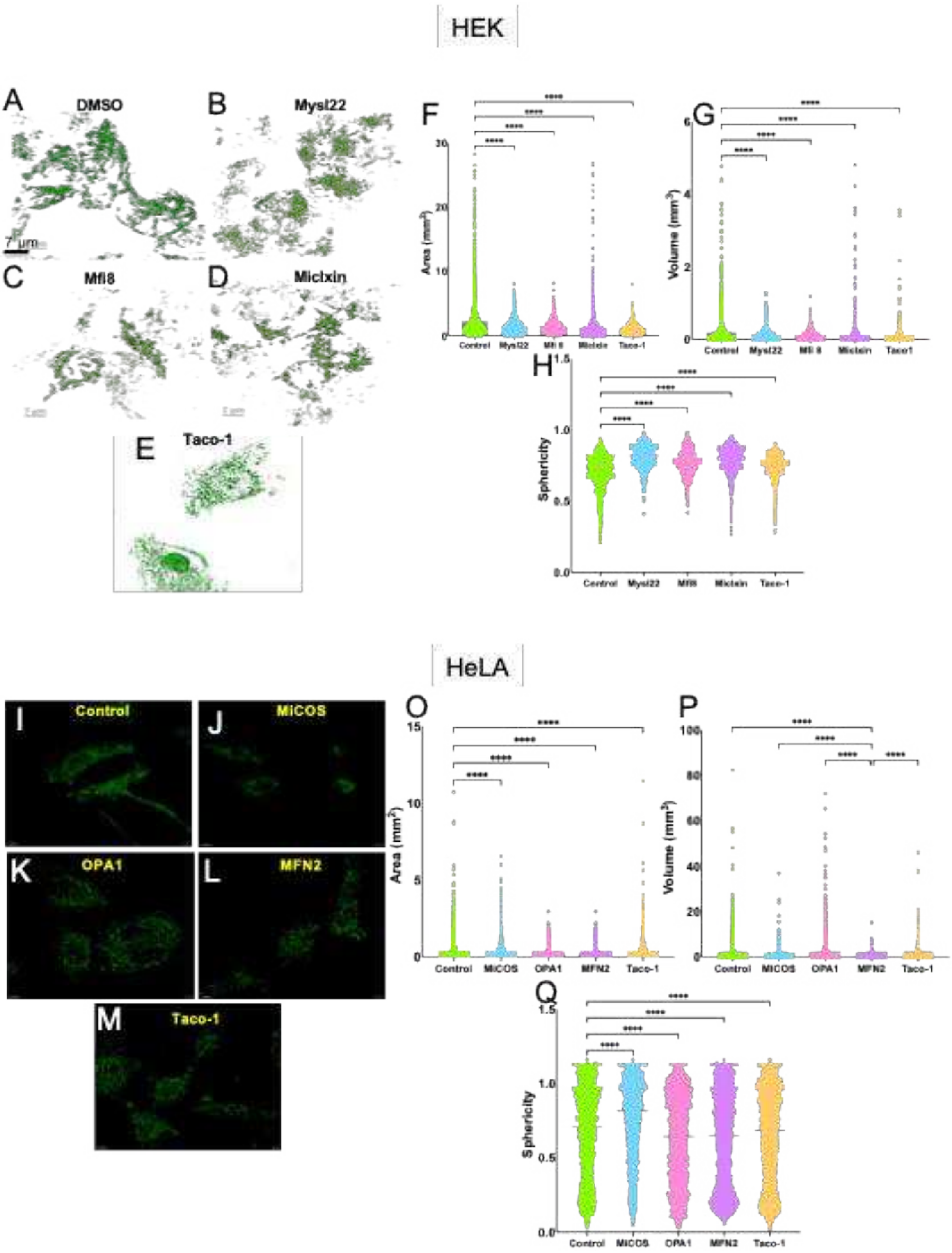

Figure S4: **TACO1 loss reduces mitochondrial area and volume while increasing sphericity in HEK and HeLa cells.** (A–E) Representative 3D-reconstructed fluorescence images of the mitochondrial network (green) in HEK cells treated with DMSO control (A), Mysl22 (B), Mfi8 (C), MiclXin (D), or Taco-1 (E). Scale bar = 7 µm. (F–H) Quantification of mitochondrial area (F), volume (G), and sphericity (H) in HEK cells across all conditions, displayed as violin plots. All treatment conditions show significant reductions in mitochondrial area and volume, and significant increases in sphericity, relative to DMSO-treated controls. (I–M) Representative 3D-reconstructed fluorescence images of the mitochondrial network in HeLa cells transfected with control (I), MiCOS (J), OPA1

(K), MFN2 (L), or Taco-1 (M) siRNA. Scale bar = 7 µm. (O–Q) Quantification of mitochondrial area (O), volume (P), and sphericity (Q) in HeLa cells across all knockdown conditions, displayed as violin plots. All knockdown conditions produced significant reductions in mitochondrial area and volume, and significant increases in sphericity, compared to control siRNA-transfected cells. Data represent individual mitochondrial measurements pooled across multiple cells and biological replicates. One-way ANOVA determined statistical significance with post-hoc multiple comparisons testing. ****p < 0.0001.

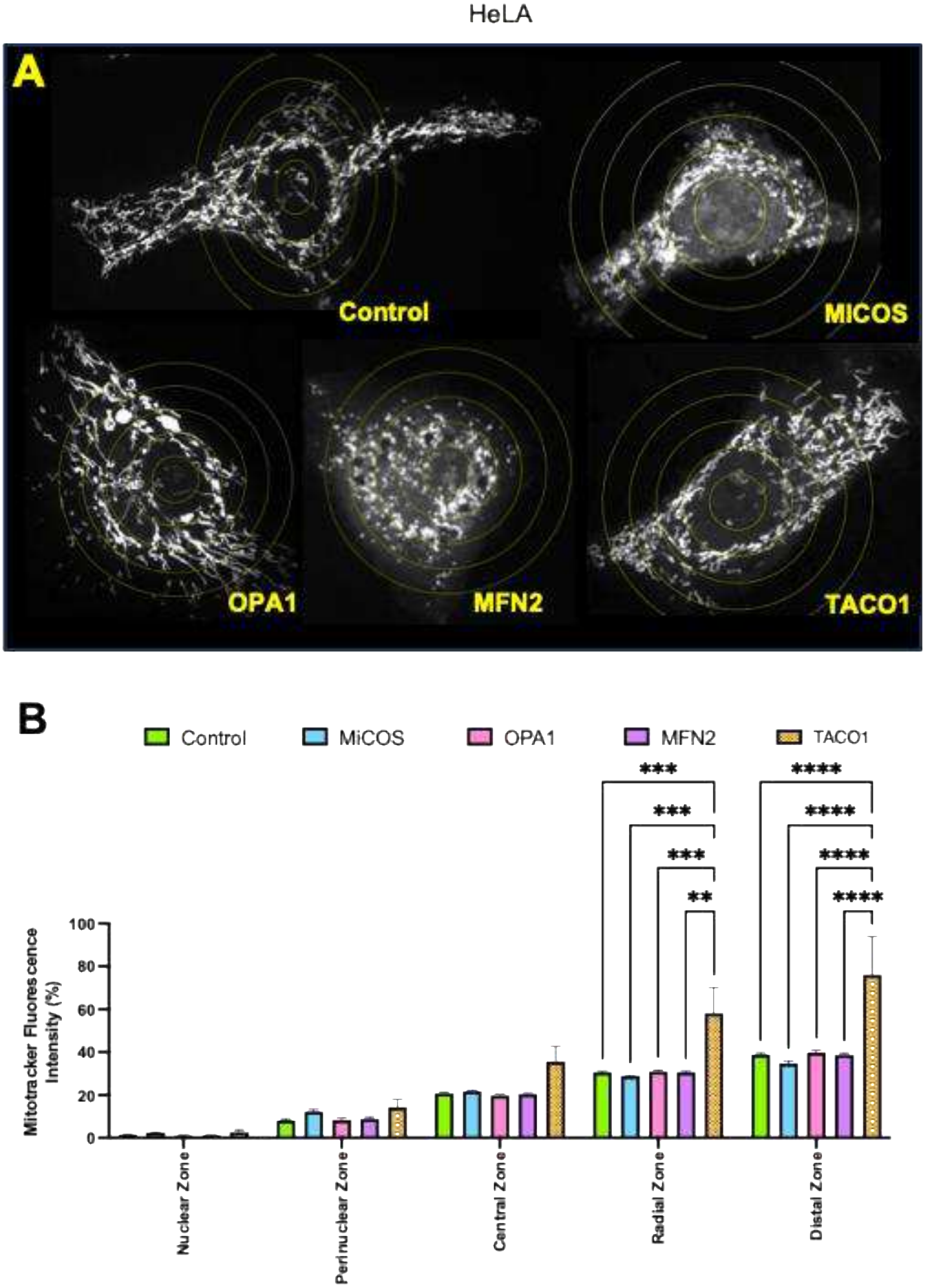

Figure S5. TACO1 knockdown drives peripheral redistribution of mitochondria in HeLA cells.

(A) Representative MitoTracker fluorescence images of HeLA cells transfected with control, MICOS, OPA1, MFN2, or TACO1 siRNA. Concentric circle overlays (yellow) are centered on the nucleus and define five radial zones used to quantify the subcellular distribution of mitochondria: nuclear, perinuclear, central, radial, and distal. (B) Quantification of MitoTracker fluorescence intensity (expressed as a percentage of total cellular signal) within each concentric zone for control (green), MICOS (blue), OPA1 (pink), MFN2 (purple), and TACO1 (orange, hatched) knockdown conditions. TACO1 depletion results in a significant shift the mitochondrial signal toward the radial and distal zones compared with all other conditions, indicating a peripheral redistribution of the mitochondrial network. Data represent mean ± SEM. One-way ANOVA determined statistical significance using one-way ANOVA with post hoc multiple comparisons. **p < 0.01, ***p < 0.001, ****p < 0.0001.

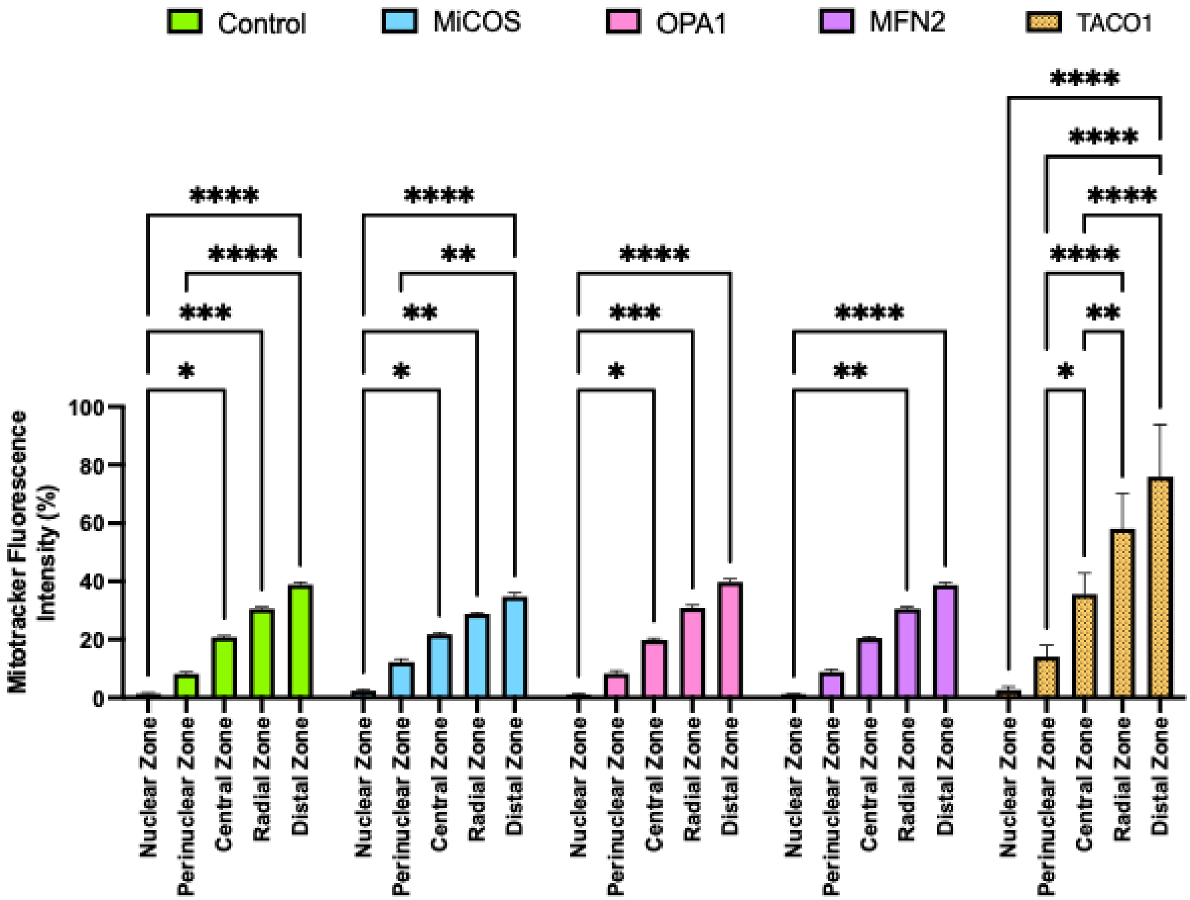

Figure S6: Peripheral redistribution of mitochondria in HeLa cells (MICOS, OPA1, MFN2, TACO1).

Table S1. Bonferroni and nominally significant circulatory and endocrine/metabolic phecodes associated with *TACO1* loss-of-function carrier status in the *All of Us* cohort. Each row represents a phecode-defined circulatory or endocrine/metabolic phenotype nominally associated with *TACO1* predicted loss-of-function carrier status in covariate-adjusted logistic regression. Carrier status was modeled as the independent variable of interest and phecode case status as the outcome, adjusting for age at last event, sex at birth, and the first five principal components. Cases and controls indicate the numbers of participants in the analytic cohort with and without the corresponding phecode. OR, odds ratio; CI, confidence interval.

Phecode	Phenotype	Category	Cases	Controls	Beta	OR (95% CI)	*p*-value
395.2	Nonrheumatic aortic valve disorders	circulatory system	5139	171762	4.321	75.262 (14.182, 399.415)	3.87e-07
430.1	Subarachnoid hemorrhage	circulatory system	498	179226	4.012	55.249 (6.673, 457.145)	1.99e-04
459.1	Hemorrhage NOS	circulatory system	625	177729	3.624	37.485 (4.522, 310.754)	7.83e-04
246	Other disorders of thyroid	endocrine/metabolic	4703	171988	2.38	10.808 (2.04, 57.283)	5.14e-03
430	Intracranial hemorrhage	circulatory system	1526	177609	2.94	18.919 (2.282, 156.805)	6.44e-03
279.11	Deficiency of humoral immunity	endocrine/metabolic	1098	178482	2.899	18.161 (2.153, 153.086)	7.69e-03
395.3	Nonrheumatic tricuspid valve disorders	circulatory system	1461	175677	2.904	18.249 (2.145, 155.4)	7.86e-03
429.3	Symptoms involving cardiovascular system	circulatory system	6518	164610	2.164	8.702 (1.697, 44.612)	9.48e-03
420.2	Pericarditis	circulatory system	1864	176445	2.724	15.234 (1.837, 126.343)	1.16e-02
244	Hypothyroidism	endocrine/metabolic	23137	152523	1.787	5.969 (1.354, 26.311)	1.83e-02
415.2	Chronic pulmonary heart disease	circulatory system	2291	176069	2.416	11.205 (1.34, 93.691)	2.57e-02
420	Carditis	circulatory system	3057	174419	2.273	9.705 (1.162, 81.126)	3.59e-02
429	Ill-defined descriptions and complications of heart disease	circulatory system	9915	159399	1.725	5.613 (1.074, 29.371)	4.10e-02
250.41	Impaired fasting glucose	endocrine/metabolic	9523	166436	1.72	5.584 (1.05, 29.666)	4.36e-02

Movie 1: Representative video of three-dimensional mitochondrial reconstructions from Serial Block-Face

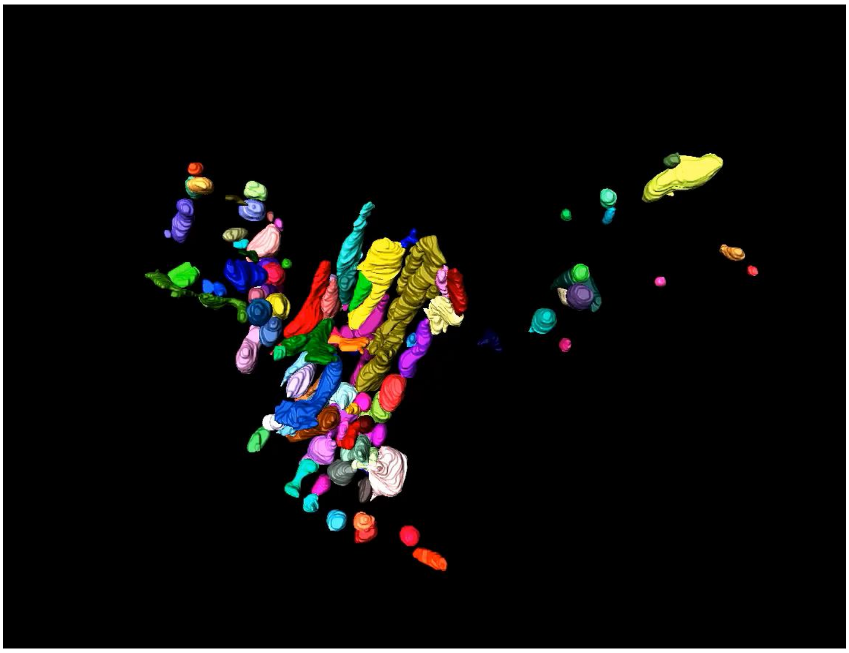

Scanning Electron Microscopy volumes of heart failure sample. Rotational visualization of mitochondrial

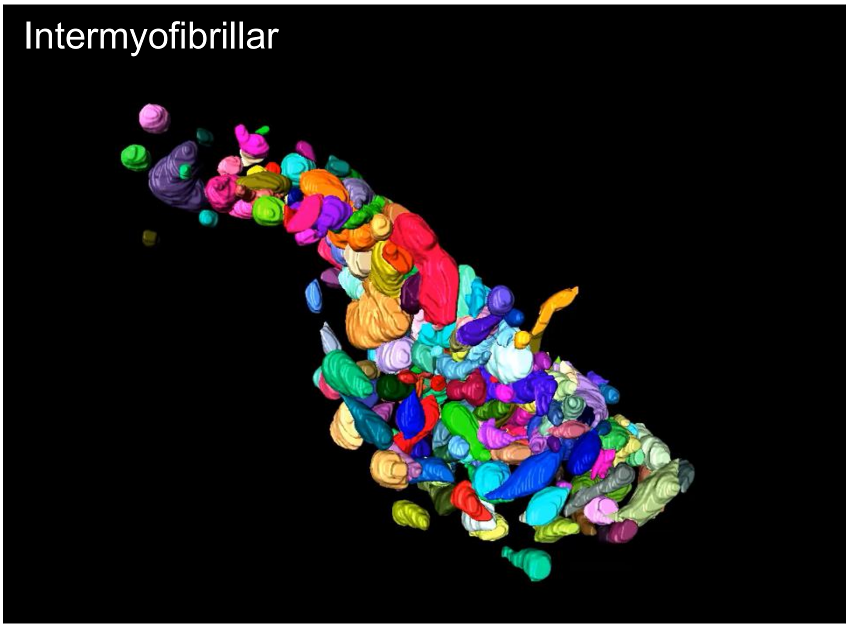

reconstruction from Subsarcolemmal in a heart failure subject.

Movie 2: Representative video of three-dimensional mitochondrial reconstructions from Serial Block-Face Scanning Electron Microscopy volumes of heart failure sample. Rotational visualization of mitochondrial reconstruction from Intermyofibrillar in a heart failure subject.

Movie 3:

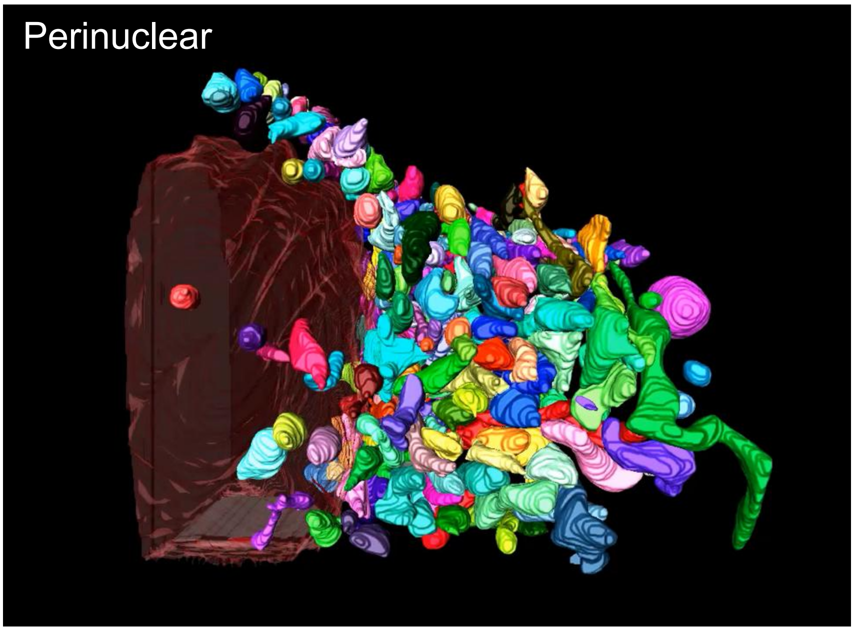

Representative video of three-dimensional mitochondrial reconstructions from Serial Block-Face Scanning Electron Microscopy volumes of heart failure sample. Rotational visualization of mitochondrial reconstruction from Perinuclear in a heart failure subject.
